# Comparison of three frailty measures for predicting hospitalization and mortality in the Canadian Longitudinal Study on Aging

**DOI:** 10.1007/s40520-024-02706-w

**Published:** 2024-02-29

**Authors:** Romain Pasquet, Mengting Xu, Marie-Pierre Sylvestre, Mark R. Keezer

**Affiliations:** 1 Centre de Recherche du Centre Hospitalier de l’Université de Montréal (CRCHUM), 1000, Rue Saint-Denis, Montréal, QC H2X 0C1 Canada; 2 School of Public Health, Université de Montréal, Montreal, QC Canada; 3 Department of Neurosciences, Université de Montréal, Montreal, QC Canada

**Keywords:** CLSA, Frailty index, Frailty phenotype, Grip strength, Predictive performance

## Abstract

**Background:**

Few studies have compared different measures of frailty for predicting adverse outcomes. It remains unknown which frailty measurement approach best predicts healthcare utilization such as hospitalization and mortality.

**Aims:**

This study aims to compare three approaches to measuring frailty—grip strength, frailty phenotype, and frailty index—in predicting hospitalization and mortality among middle-aged and older Canadians.

**Methods:**

We analyzed baseline and the first 3-year follow-up data for 30,097 participants aged 45 to 85 years from the comprehensive cohort of the Canadian Longitudinal Study on Aging (CLSA). Using separate logistic regression models adjusted for multimorbidity, age and biological sex, we predicted participants' risks for overnight hospitalization in the past 12 months and mortality, at the first 3-year follow-up, using each of the three frailty measurements at baseline. Model discrimination was assessed using Harrell’s c-statistic and calibration assessed using calibration plots.

**Results:**

The predictive performance of all three measures of frailty were roughly similar when predicting overnight hospitalization and mortality risk among CLSA participants. Model discrimination measured using c-statistics ranged from 0.67 to 0.69 for hospitalization and 0.79 to 0.80 for mortality. All measures of frailty yielded strong model calibration.

**Discussion and conclusion:**

All three measures of frailty had similar predictive performance. Discrimination was modest for predicting hospitalization and superior in predicting mortality. This likely reflects the objective nature of mortality as an outcome and the challenges in reducing the complex concept of healthcare utilization to a single variable such as any overnight hospitalization.

**Supplementary Information:**

The online version contains supplementary material available at 10.1007/s40520-024-02706-w.

## Introduction

As people age, they become more vulnerable to declining health status, increasing the risk of dependency, institutionalization, and mortality [1]. Such vulnerability varies among people of the same age and this is referred to as frailty [1–3]. Frailty is conceptually defined as an increase in vulnerability as a result of an aging-associated decline in reserve and function across multiple physiological systems. This decline limits a person’s ability to cope with everyday or acute stressors [3]. There are many symptoms associated with frailty and frailty can occur with or without specific diseases. Multimorbidity is often present among frail individuals, either as a consequence or a cause of frailty [2, 4]. Numerous studies have demonstrated that frailty is associated with adverse health outcomes including increased risk of mortality, hospitalization, disability, falls, delirium, and admission to long-term care [1, 5–11]. As populations in high-income countries continue to age, frailty has emerged as an important health concern, with major implications for public health and clinical practice [12]. The estimated prevalence of frailty varies greatly, from 12 to 24% among individuals aged ≥ 50 years across over 60 countries, likely due to differences in the methods used to measure frailty and in the characteristics of the studied populations [13]. It has been a theoretical challenge to define the concept of frailty and to operationalize it [14]. Although progress has been made over the past decades, there is still no consensus on the operational definition of frailty and no standardized measure has been developed [12].

Previous studies have attempted to operationalize measures of frailty using various methods, falling within the following two common approaches: the phenotype approach [1] and the frailty index (FI) approach [15]. The phenotype approach conceptualizes frailty as a biological syndrome of decreased reserve and resistance to stressors, which results from cumulative declines across multiple physiologic systems [1]. Under this biological syndrome model, frailty is measured using five components: muscle weakness, unintentional weight loss, exhaustion, slow walking speed, and low activity level. Participants are classified as frail if their performance is poor on three or more of the above criteria [1]. The FI approach employs a measure of cumulative burden of symptoms, diseases, conditions, and disability [15]. This approach measures frailty as the proportion of age-related health and functional deficits in individuals, including psychological, social, and environmental factors of health, out of a total number of measured health conditions (generally a minimum of 30) [15]. Grip strength is also presented as a measure of frailty, representing what is hypothesized to be a central aspect of the frailty phenotype [16, 17]. This is based on the observation that grip strength, when compared to chronological age, is correlated with a greater number of markers of frailty including cognitive function, lens opacity, and number of teeth [16]. Moreover, reduced grip strength has also been shown to be an important predictor of disability, morbidity, hospitalization, and mortality in middle-aged and older populations [17–19].

Few studies have compared these different measures of frailty for predicting adverse outcomes [20]. One study compared two FIs and concluded that both are predictive of key geriatric outcomes [21], although it remains uncertain which frailty measurement approach best predicts healthcare utilization such as hospitalization, and mortality [22–24]. Healthcare utilization is a useful means of studying the overall health of an individual or population. As opposed to mortality, healthcare utilization is not a terminal event, leaving room for continued intervention.

To better inform research at the level of the general population, as well as public health decision making, it is important to identify which frailty measure best predicts healthcare utilization and mortality so that individuals who are frail can be identified early and appropriate interventions can be implemented to reduce the likelihood of adverse health consequences [25]. This study aims to compare three approaches to measuring frailty—grip strength, frailty phenotype, and FI—in predicting hospitalization and mortality among middle-aged and older Canadians.

## Methods

### Study population

In this study, we assessed how different frailty measures predict overnight hospitalization and mortality using data from the Canadian Longitudinal Study on Aging (CLSA). The CLSA is a national, longitudinal research platform which aims to examine and address the needs of the aging Canadian population [26].

The full CLSA cohort includes 51,338 people from all 10 Canadian provinces, aged 45 to 85 years at the time of recruitment. This full cohort is composed of two sub-cohorts: (1) the tracking cohort comprised of 21,241 participants randomly selected from within the 10 provinces who are interviewed by telephone, and (2) the comprehensive cohort comprised of 30,097 participants randomly selected from within 25–50 km of 11 data collection sites (available in seven provinces). Participants in the comprehensive cohort are interviewed in person, undergo in-depth physical assessments at the data collection sites, and provide blood and urine samples. Persons living on federal First Nation reserves or working as full-time members in the Canadian Armed Forces, and persons with cognitive impairment at the time of recruitment, who were institutionalized, or unable to communicate in English or French were excluded from the study. Recruitment and baseline data collection were completed in 2015 and the first follow-up was completed in 2018.

Detailed descriptions of the selection and recruitment processes have been published [26, 27]. The participation rate into the CLSA was approximately 45% among those who provided their contact information for study recruitment and the overall response rate was 10%. All participants provided written informed consent.

The present analysis used data of the 30,097 participants from the CLSA comprehensive cohort, for whom data were available to construct the different measures of frailty. Participants with contraindications to perform certain tests or measurements required to measure frailty were excluded from the analysis. A flow chart of CLSA participants who met the inclusion/exclusion criteria for the present study can be found in Supplementary Fig. 1.

### Frailty assessments

Using three approaches, we assessed frailty at baseline: grip strength, frailty phenotype, and FI.

For the grip strength approach, the average grip strength for each participant measured at baseline was used as the only indicator of frailty. The dominant hand grip strength, unless contraindicated, was measured three times for each participant using the Tracker Freedom® Wireless Grip Dynamometer in a straight-backed chair. Contraindications for grip strength measurement included surgery on both hands or wrists within the last three months; pain or paralysis in both hands or wrists due to arthritis, tendinitis, carpal tunnel syndrome; cast on both arms or hands; open sores, wounds or bruising on both hands; and prosthetic arms, hands or fingers on both sides [28]. Grip strength weakness was assessed both continuously (in kilograms) and categorically (weak = lowest quintile, stratified by sex [biological sex assigned at birth] and body mass index [BMI] class; not weak = all other values) in our analyses. In addition, we conducted sensitivity analyses replacing average grip strength with max grip strength (see Supplementary Table 1).

For the phenotype approach, the operationalization [1] proposed by Fried et al. was applied to determine participants' frailty status by using five criteria to assess the presence/absence of signs and symptoms central to frailty. These are muscle weakness, weight loss, exhaustion, slowness, and low physical activity. Our operationalization of the frailty phenotype was based upon prior work using the CLSA [29]. Muscle weakness was defined as the average grip strength over all grip strength trials of participants. Weight loss was defined as pounds lost over the last six months. Exhaustion was defined by a participant’s responses to two questions: "How often did you feel that you could not ‘get going’?" and "How often did you feel that everything you did was an effort?". Slowness was measured as the total time required to complete the 4-m walk (in seconds). Low physical activity was measured using the score calculated from the Physical Activity Scale for the Elderly (PASE) questionnaire [30]. To classify the presence or absence of an abnormal frailty phenotype, we dichotomized each phenotype component into abnormal vs. normal using previously published thresholds [29]. A detailed description of the cut-off criteria can be found in Supplementary Table 2. Based on the absolute count of abnormal phenotype components for each participant, we assessed frailty both as an ordinal variable (the number of abnormal components of phenotype) and as a categorical variable (not frail: less than three abnormal phenotype components, frail: three to five abnormal phenotype components).

For the FI approach, we adopted Rockwood’s deficit accumulation measures [15] to assess baseline frailty status through the calculation of a FI value for each participant. Ninety-three deficit variables related to chronic health, functional status, activities of daily living, mental health, nutritional risk, physical activities, and perceived health were considered in the FI calculation. These variables were selected based on a previous publication that aimed to adapt the FI to the CLSA [14]. A detailed description of the 93 variables is found in Supplementary Table 3. All deficit variables were categorized dichotomously, with a value of 1 denoting the presence and a value of 0 denoting the absence of the deficit in question. The FI value was calculated as the sum of deficits present divided by the total number considered. The FI was assessed both as a continuous variable (with values ranging from 0 to 1.0) and as a categorical variable (not frail = FI ≤ 0.20; frail = FI > 0.20).

### Outcome assessments

CLSA participants were recontacted during the first follow-up 3 years after the initial data collection interview. Self-reported occurrence of any overnight hospitalization not including emergency department visits in the preceding 12 months (yes/no) was ascertained in questionnaires administered to the participants during computer-assisted interviews, and it was analyzed as a binary outcome variable. Mortality (yes/no) was ascertained using data on death confirmed by health ministry or other sources. Participants who died after recruitment, but still participated in the first follow-up 3 years after the initial data collection, were considered alive at this first follow-up since the exact time to death was not known.

### Statistical analysis

#### Correlation between each measure of frailty

The Phi correlation coefficients were used to estimate correlations between categorical versions of frailty measurements. We also calculated the percent agreement between them.

We used regression models to estimate the strength of the association between each measure of frailty, and hospitalization as well as mortality.

Using each of the frailty measures (grip strength, frailty phenotype, and FI), multivariable logistic regression models were used to predict overnight hospitalizations and mortality. The comprehensive cohort analytic weights were used for the regression analyses [31]. These weights aim to correct the distribution of age, sex, province, whether living near a data collection site or not, and level of education, so that these better match the general Canadian population. Each of the frailty measures was analyzed categorically and continuously. We evaluated the linearity of the relationship between the selected continuous frailty measures and overnight hospitalization and mortality by categorizing the continuous version of each frailty measure into four categories based on the quartiles among controls, calculating the odds ratios and 95% confidence intervals (95% CIs) for each categorical measure, hospitalization, and mortality, showing the odds ratios in a plot. Because a majority of participants had a value of 0 for the phenotype approach, we categorized them into four categories based on the number of present phenotype components (i.e., 1: no phenotype component, 2: one frailty component, 3: two phenotype components and, 4: three or more phenotype components). Missing data were handled using multiple imputation with 15 iterations based on nearly 200 auxiliary variables (see full list of variables and their definitions in Supplementary Tables 1 to 3).

To explore whether predictive performance differs due to potential biological sex differences or age in the relationship between frailty and our selected outcomes, we conducted sensitivity analyses stratifying by sex and restricting to participants aged 65 years and older. To simplify comparisons between the frailty measures, as well as to allow the combining of grip strength data between males and females, we standardized the continuous measures of frailty using the z-score method. It is important to note that higher values in the continuous frailty phenotype and index variables are associated with a higher probability of frailty, while the opposite is true for higher grip strength. Consequently, opposite associations are expected to be observed between those analyses.

#### Covariate adjustments

Models were all adjusted for multimorbidity, age, and biological sex. The multimorbidity covariate was assessed using a multimorbidity index, calculated as an absolute count of a lifetime history of chronic conditions (Supplementary Table 4) present in the CLSA participants [32]. We also conducted sensitivity analyses adjusting only for age and biological sex.

#### Model discrimination and calibration

We evaluated the predictive performance of each model by assessing model discrimination and calibration. Model discrimination was assessed using Harrell’s c-statistics (95% CI), and model calibration was compared through visual examination of calibration plots with loess smoothers [33, 34]. Model discrimination measures the extent to which a model can predict a higher probability of having an outcome among subjects having that outcome versus those not having it. For binary outcomes, Harrel’s c-statistic is often used to measure discrimination. A c-statistic value of 0.5 indicates that a model discriminates no better than chance between participants with and without the outcome, and a c-statistic value of 1.0 indicates that a model consistently assigns a higher probability of having an outcome for participants with the outcome versus those without it. A generally accepted approach suggests that a *c*-statistic of < 0.60, 0.60 to 0.75, and > 0.75 reflects poor, possibly helpful, and clearly useful discrimination, respectively [33]. Model calibration measures the extent to which a model’s predicted values agree with the observed values. Calibration plot allows for a visual examination of the relationship between the predicted and the observed outcomes for model calibration.

All statistical analyses were conducted using R version 4.0.0. The “mice” package was used to conduct the multiple imputation, and the “psfmi” package was used to examine the pooled performance of each model.

## Results

Of the 30,097 participants from the CLSA comprehensive cohort, we excluded 1456 participants who were unable to complete the grip strength assessment and 113 participants who were unable to perform the 4-min walk assessment due to contraindications, and one participant who was pregnant at the time of the baseline interview and thus was unable to be assigned a BMI class. This led to a total of 28,527 participants included in the analyses (Supplementary Fig. 1). Selected characteristics of study participants in the unimputed and pooled imputed dataset are presented in Supplementary Table 5. Participant mean age was 62.8 years (standard deviation [SD]: 10.2) and 50% of participants were of female sex. In the pooled imputed dataset, the proportion of participants classified as frail using the average grip strength, max grip strength, FI, and frailty phenotype were 20.0%, 20.0%, 8.1% and 6.0%, respectively. The mean value for the multimorbidity index was 4.0 (SD: 2.4) while 9.2% of participants were hospitalized and 1.8% were dead. Similar results were observed in the unimputed dataset. All associations were linear based on the quartiles analyses (Supplementary Figs. 2 and 3).

We calculated the correlations between the categorical versions of the frailty measurements, as well as the percent agreement between them. Frailty measured using the average grip strength was very strongly correlated with frailty measured using the max grip strength (correlation coefficient = 0.89). Average grip strength was moderately correlated with the frailty phenotype approach (correlation coefficient = 0.39), and weakly correlated with the FI approach (correlation coefficient = 0.19). Frailty measured using the frailty phenotype approach was moderately correlated with the FI approach (correlation coefficient = 0.38). Frailty measured using average grip strength had a percent agreement of 96.6% with max grip strength, 83.9% with the frailty phenotype, and 79.5% with the FI. Frailty measured using the frailty phenotype had a percent agreement of 92.2% with the FI.

**Fig. 1:**
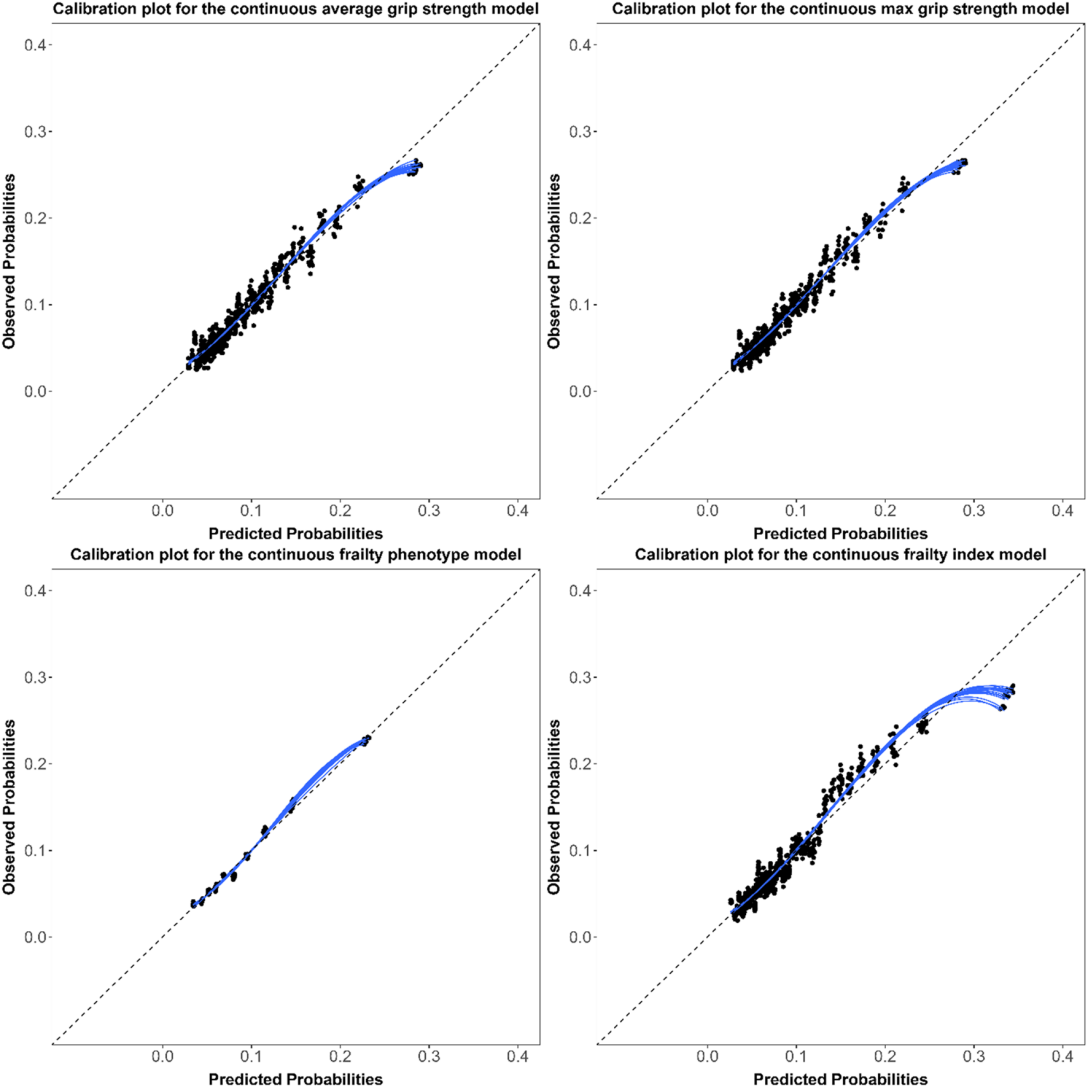
Calibration plots for the continuous hospitalization models in both sexes combined

**Fig. 2:**
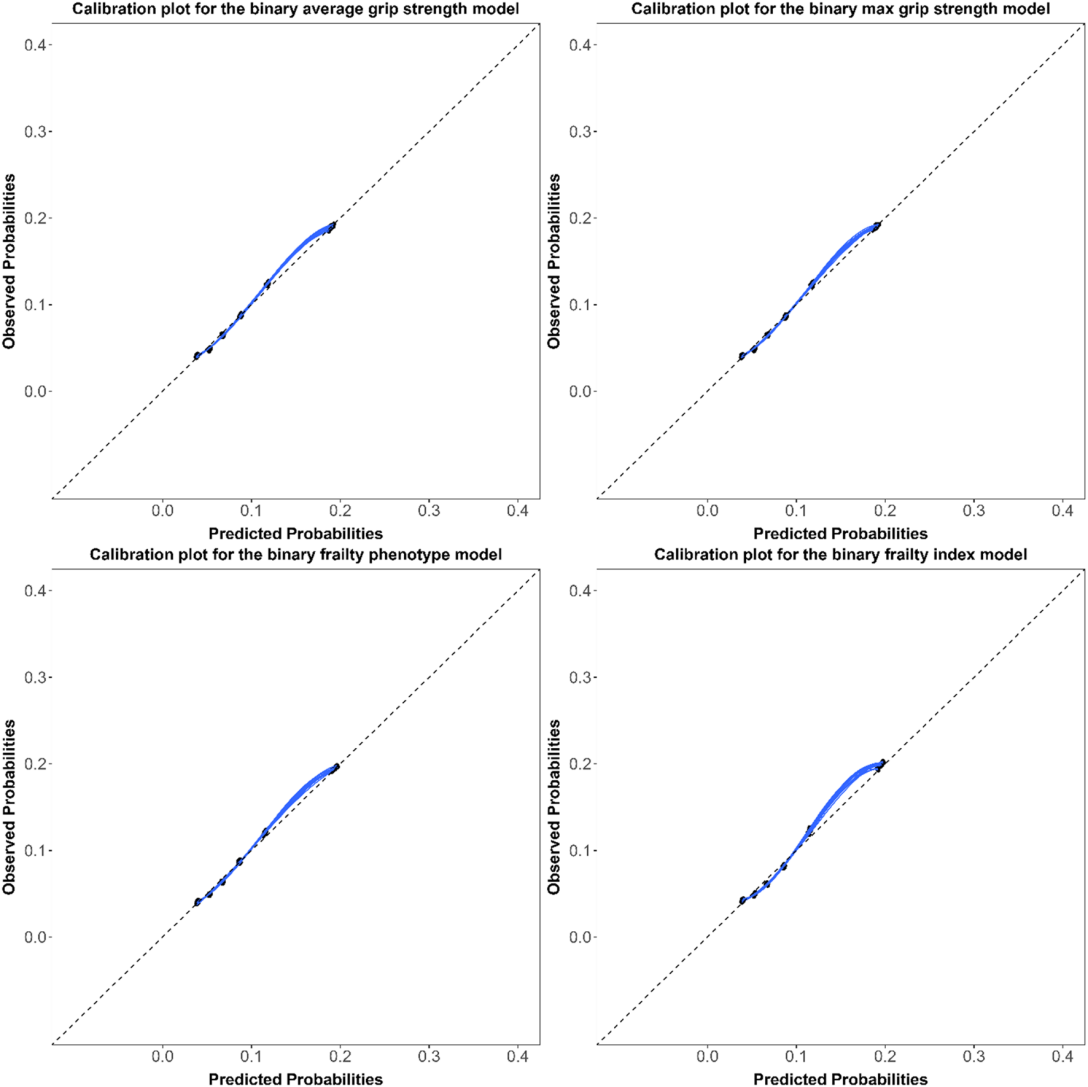
Calibration plots for the binary hospitalization models in both sexes combined

**Fig. 3:**
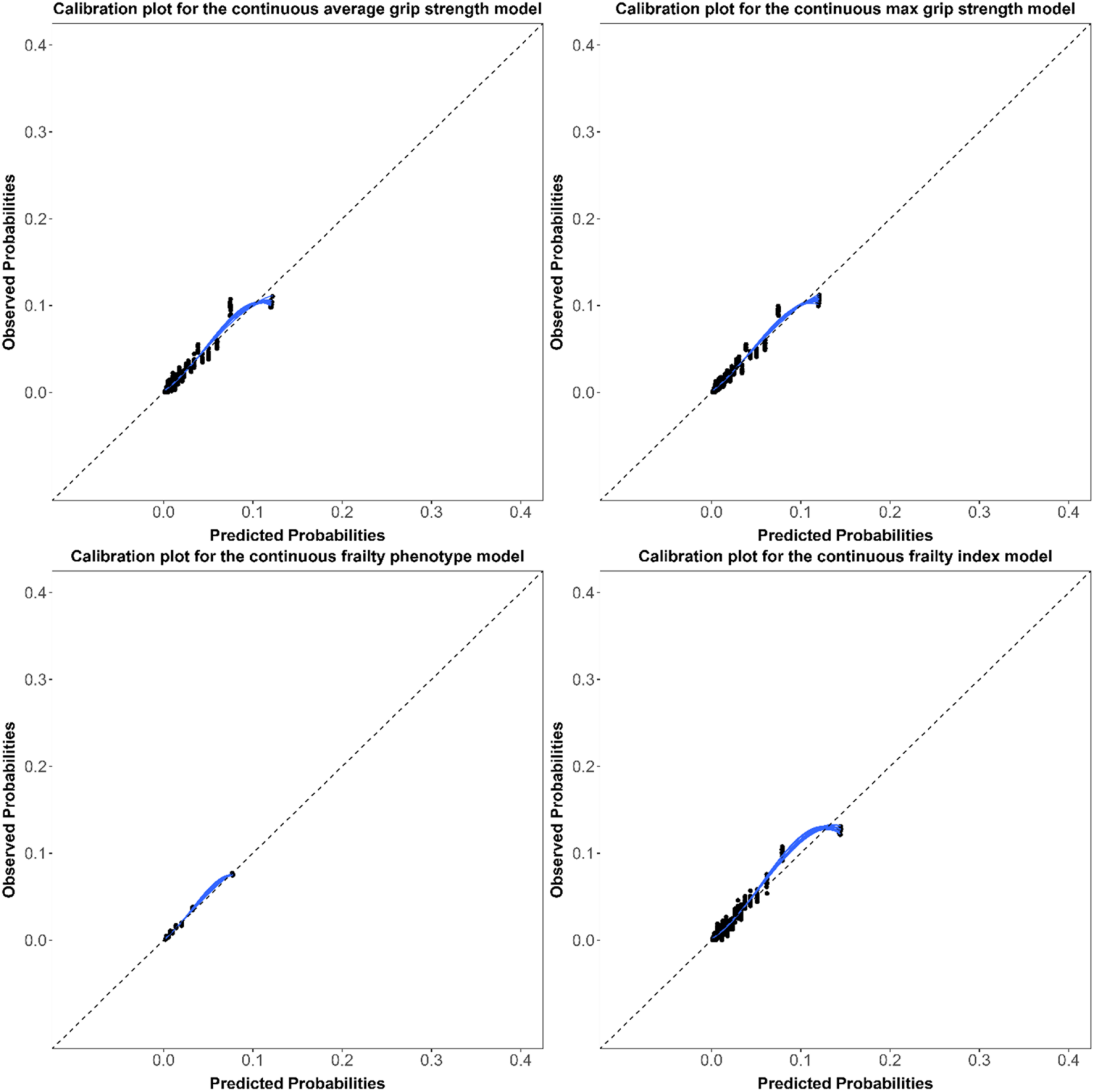
Calibration plots for the continuous mortality models in both sexes combined

**Fig. 4:**
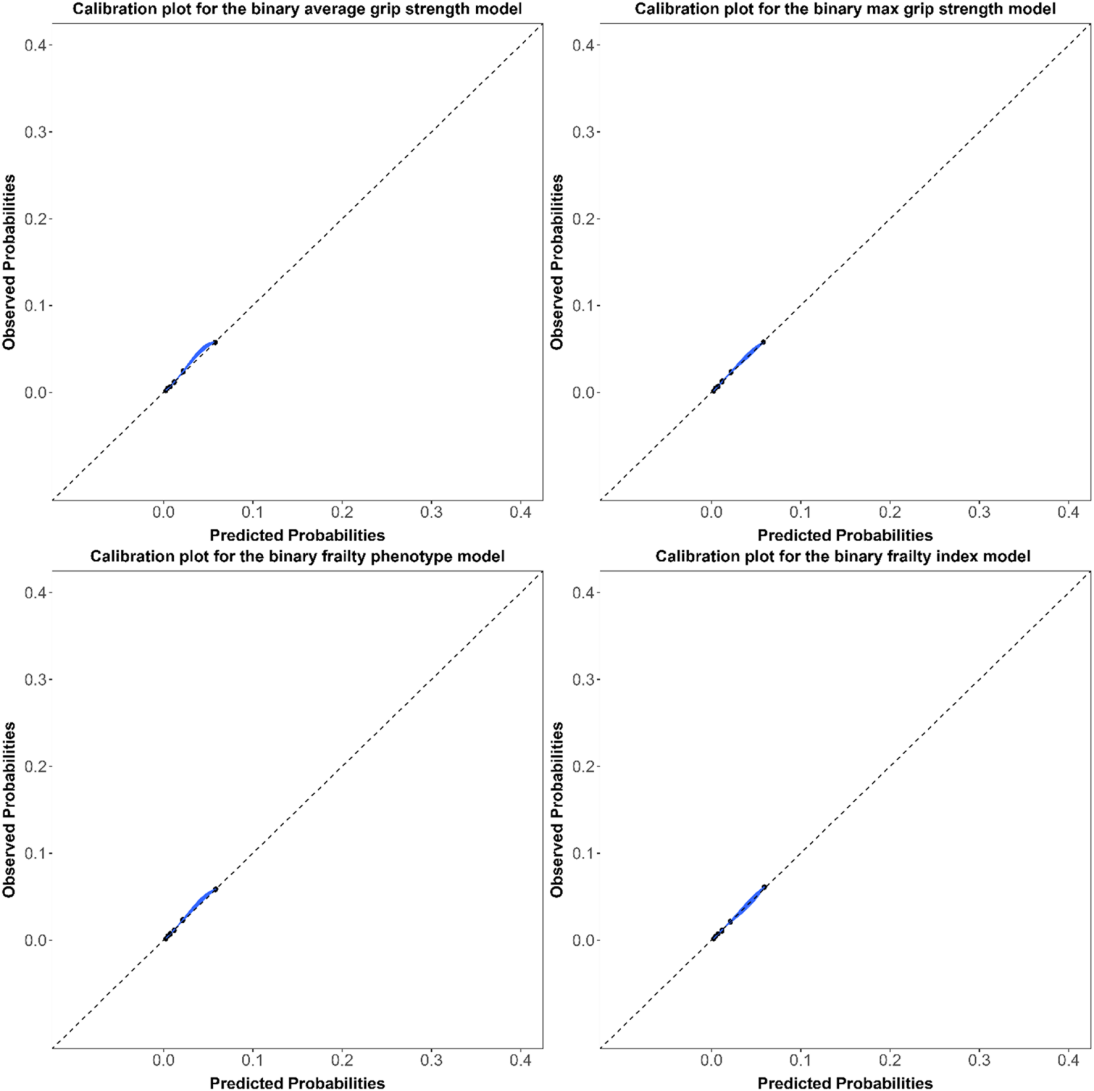
Calibration plots for the binary mortality models in both sexes combined

Model discrimination assessed using the c-statistics (95% CIs) were similar for all hospitalization models (Table 1), ranging from 0.67 (0.66, 0.68) to 0.69 (0.68, 0.70). The corresponding c-statistics were 0.11 to 0.12 points higher in the mortality models, ranging from 0.79 (0.77, 0.80) to 0.80 (0.79, 0.82). Models were overall well-calibrated with little meaningful differences between models (Figs. 1, 2, 3, 4). For the continuous versions of the FI and both grip strength variables, calibration was poorer when the predicted probabilities were > 0.3 in the hospitalization models and > 0.1 in the mortality models, in addition, the calibration curves for both outcomes did not span from 0 to 1.

**Table 1:** Adjusted ORs (95% CIs) and c-statistics (95% CIs) for the association between different indicators of frailty in older adults and hospitalization or mortality in both sexes combined

Frailty indicators	Hospitalization		Mortality	
	OR (95% CI)	C-statistic (95% CI)	OR (95% CI)	C-statistic (95% CI)
*Grip strength*				
Average				
Continuous^a,b^	0.95 (0.88–1.02)	0.67 (0.66- 0.68)	0.63 (0.53–0.75)	0.79 (0.78–0.80)
Frail versus non-frail^c^	1.04 (0.92–1.17)	0.67 (0.66 -0.68)	1.49 (1.17–1.90)	0.79 (0.77–0.80)
Max				
Continuous^a,b^	0.93 (0.86–1.01)	0.67 (0.66–0.68)	0.62 (0.52–0.74)	0.79 (0.77–0.80)
Frail versus non-frail^c^	1.06 (0.94–1.20)	0.67 (0.66–0.68)	1.58 (1.24–2.02)	0.79 (0.77–0.80)
*Frailty phenotype*				
Continuous^a,d^	1.23 (1.17–1.28)	0.68 (0.67–0.69)	1.77 (1.61–1.94)	0.80 (0.78–0.81)
Frail versus non-frail^e^	1.66 (1.40–1.95)	0.67 (0.66–0.69)	3.29 (2.47–4.38)	0.79 (0.78–0.80)
Frailty index				
Continuous^a,d^	1.46 (1.38–1.54)	0.69 (0.68–0.70)	1.87 (1.68–2.09)	0.80 (0.79–0.82)
Frail versus non-frail^f^	1.83 (1.56–2.14)	0.68 (0.66–0.69)	3.33 (2.48–4.47)	0.79 (0.78–0.81)

*OR* odds ratio, *CI* confidence interval

Models were adjusted for the multimorbidity index, age, and sex

^a^Standardized using the z-score method

^b^Lower values for grip strength indicates higher degree of frailty

^c^Frail: Lowest quintile stratified by sex and BMI class. Non-frail: All other higher quantiles

^d^Higher values for the frailty phenotype and index indicate higher degree of frailty

^e^Frail: 3 or more of the five phenotype components present. Non-frail: 0–2 of the five phenotype components present

^f^Frail: FI > 0.2. Non-frail: FI ≤ 0.2

We observed broadly similar results when examining the c-statistics by biological sex (Supplementary Tables 6 and 7), albeit the c-statistics were consistently 0.02–0.03 higher in males as compared to females. The calibration plots were also similar (Supplementary Figs. 4 to 11) except for an overall slightly poorer calibration in models examining the prediction of mortality by the binary version of the frailty measures in females (Supplementary Fig. 11). This was most noticeable for the FI model. When restricting the analyses to participants aged 65 and older (Supplementary Table 8), we found no difference in the predictive performance of each of the three measures of frailty and hospitalization. The c-statistic for each of three measures of frailty, however, dropped by 10 to 15% for mortality, indicating that our predictive models perform better or the same when including both middle-aged and older adults, depending on the selected health outcome. We also found slightly worse model calibration when omitting the multimorbidity index in the covariate adjustment (Supplementary Table 9).

Frailty measured using any of the selected measurements predicted an increased risk of hospitalization and mortality. All measures more strongly predicted mortality when compared to hospitalization (Table 1). GS, both average and max, less strongly predicted hospitalization and mortality, as compared to the frailty phenotype and FI. Similar results were observed with the complete case analysis using the unimputed dataset (Supplementary Table 10). When stratifying by biological sex (Supplementary Tables 6 and 7), we also observed similar results to the main findings presented in Table 1. An exception were the predictions of mortality by average and max grip strength, where there were strong trends for a stronger prediction for males as compared to females, although the 95% CI were generally wide with corresponding overlap.

## Discussion

In this study, we measured frailty using three approaches (grip strength, frailty phenotype, and the FI) using CLSA data, a large Canadian general-population longitudinal study of middle-aged and older participants. We examined the prediction of hospitalization and mortality at the 3-year follow-up by frailty measured at baseline. We compared the predictive performance of each frailty measure for each outcome. The grip strength approach classified the most participants as frail (20%), followed by the FI approach (8%) and finally by the frailty phenotype approach (6%). Our study found moderate correlations between the grip strength and phenotype approaches, and between the phenotype and FI approaches for the identification of people with frailty. We also found a weak correlation between the grip strength approach and the FI approach. The present analysis showed that all three measures of frailty were equally useful predictors for overnight hospitalization and mortality among participants in the CLSA cohort. Model discrimination was modest for all measures of frailty when predicting overnight hospitalization (0.67–0.69), but stronger to an important degree when predicting mortality (0.79–0.80). All measures of frailty yielded strong model calibration, although the calibration curves for both outcomes did not span from 0 to 1.

Previous studies have examined various measures of frailty, mostly among older populations [20]. Based on a review from Bouillon et al. [20], a majority of population-based studies used the phenotype approach to measure frailty, and the second most commonly used was the FI approach. The predictive validity, however, of these frailty measures for adverse health outcomes have rarely been examined in the literature [20]. One study examined the ability of the frailty phenotype, the FI, and two other measures of frailty to predict mortality, hospitalization, and dependency in activities of daily living among 2420 Dutch community-dwelling people older than 65 who were pre-frail or frail according to the frailty phenotype. The study authors concluded that all four frailty measures performed poorly in predicting the selected health outcomes in their study population [22]. Similar findings were reported in a study examining frailty measures including the frailty phenotype and FI, and their predictive performance for mortality and hospitalization among 2087 Australians aged 70 and older [23]. One study, however, that examined the predictive performance of the frailty phenotype and FI for mortality among people 50 years and older from eleven European countries reported better predictive values [24] than the studies mentioned above which only included older and more frail individuals [22–24]. Similar to this European study, the three frailty measures examined in our analysis among people aged 45 and older also had better performance in predicting health outcomes including mortality and hospitalization as compared to studies that only included older populations. When restricting our analysis to people aged 65 and older, we found no difference for the predictive performance of each of three measures of frailty and hospitalization. The c-statistics for all three measures of frailty dropped for mortality indicating that our predictive models perform better when including both middle-aged and older populations.

The concept of frailty is increasingly used in research and clinical care. Its translation into public health interventions and clinical practice remains a challenge. Validating the predictive performance of different measurements of frailty for different health outcomes is essential. In our study, we purposely chose three measures of frailty requiring varying levels of clinical information and with overlapping predictor variables: a minimalist model using just muscle weakness as the sole indicator (the grip strength approach), a model using the five phenotype components (the frailty phenotype approach), and a comprehensive model including as much information on different aspects as frailty as possible (the FI approach). Although collecting different aspects of frailty might be less of an issue in a research setting, this is often not feasible in clinical settings where clinicians have limited time and resources. Easily employed measures are needed to facilitate the evaluation of frailty. Therefore, we wanted to examine whether adding additional aspects to define frailty would improve its predictive performance for different health outcomes. Our findings suggest that the choice of frailty measure when aiming to predict hospitalization or mortality can be based on the resources and preferences of the healthcare practionner or researcher as all three measures showed similar predictive performance.

The findings of our study are in line with previous meta-analyses examining the prediciton of hospitalization [8, 25] and mortality [35, 36] among older adults living in community-dwelling settings. Higher risks for both outcomes were found for frail individuals when compared to non-frail individuals. Our analysis adds to these prior studies by directly comparing the performance of different measures of frailty when predicting hospitalizations and mortality, all in the same study population. The weaker discrimination seen when predicting hosptilization as compared to mortality emphasizes the importance of a carefully defined and measured outcome.

Prior studies have suggested biological sex as a potential effect modifier for associations between frailty and adverse health outcomes. Studies have reported a male–female health survival paradox between the FI and mortality [37, 38]. This is that males on average have a lower FI burden, but a geater increase in the risk of mortality for each increase in FI as compared to females [38–40]. Prior studies have suggested that these sex differences may be related to sociocultural and biological factors [37]. In our analysis, there was no evidence of a difference in the strength of the prediction of hospitalization between males and females. There were modest differences, however, between average and max grip strength and mortality, with trends towards stronger odds ratios observed in males as compared to females. Additionally, we found no significant difference in the predictive performance for model discrimination or calibration in analyses of males and females.

Our study has several strengths. The CLSA is a large population-based cohort with detailed information on multiple aspects of health that afforded us the opportunity to construct different measures of frailty and evaluate their predictive performance against two health outcomes. Previous studies examining frailty often only included older adults aged 65 years and over, but frailty can also affect late middle-aged adults [41, 42]. Our study was able to include middle-aged adults in addition to older adults and demonstrates the utility of using frailty as a predictor of hospitalization and mortality among people in both age groups. In addition, we conducted multiple imputation which allowed the use of all available data to produce unbiased estimates.

Our study is not without limitations. The construction of the FI and the multimorbidity index used self-reported information on multiple chronic diseases. There is a possibility for misclassification bias due to inaccurate reporting for ascertainment of diseases. Previous validation study suggest that the self-report of chronic diseases is generally fairly accurate, except for atherosclerosis and arthritis [43]. It was also found that males tend to overreport stroke and underreport malignancies and arthritis, while females tend to overreport these conditions. The prevalence of both overreporting and underreporting of chronic diseases increase as people age [43]. For the grip strength approach, we used the average grip strength measured at baseline as a single predictor of frailty. Although this approach is less commonly used than the phenotype or the FI approach in the geriatric literature, it is often used in studies of cardiovascular diseases as a marker for frailty [44]. In our study, we excluded 5% of our participants who were unable to perform the grip strength test or the 4-min walk assessment due to medical contraindications. Additional descriptive sensitivity analyses (Supplementary Table 11) comparing participants with and without these medical contraindications revealed that, participants with medical contraindications tended to be slightly older, more likely to be of male sex, more frail, and have more comorbidities. We used overnight hospitalization within the last 12 months of the first follow-up as a proxy for healthcare utilization. A person with this outcome could have had multiple hospital stays, a single but prolonged stay, or a single short night stay. It is reasonable to hypothesize that frailty might be more strongly associated with more severe health conditions requiring more intense or frequent healthcare utilization and less so for a single night stay at the hospital. In this case, the direction of this information bias would be towards the null. In addition, since hospitalization was self-reported by participants, there is a possibility of information bias. It is difficult to predict the direction of this bias as participants can either over- or under-report their hospitalization history. Similar to many other population-based studies, the participation rate into the CLSA was low, therefore, there is a potential for selection bias due to non-response. A previous investigation of the representativeness of study participants in the CLSA study indicates that respondents may not be fully representative in terms of ethnic diversity in Canada and that they are more likely to be of higher socioeconomic status than the general population [45]. We incorporated the comprehensive cohort analytic weights, however, which aim to correct for the stratified sampling inherent to the CLSA to increase the representativeness of the sample across the general Canadian population and to mitigate the impact of non-response.

## Conclusion

All three measures of frailty had similar predictive performance for overnight hospitalization and mortality among CLSA participants. Model discrimination was consistently superior when predicting mortality as compared to hospitalization. This likely reflects the objective nature of mortality as an outcome and the challenges in reducing the complex concept of healthcare utilization to a single variable such as any overnight hospitalization. The choice of frailty measure can be based on the resources and preferences of the healthcare practitioner or researcher. It remains important to validate the predictive performance of these different measures of frailty with respect to other health outcomes such as living in an assisted living environment.

### Supplementary Information

Below is the link to the electronic supplementary material.

Supplementary file1 (DOCX 885 KB)

## Data Availability

An access request may be submitted to the CLSA to obtain the data used in the current secondary analysis (https://www.clsa-elcv.ca/data-access).
