# Supplementary material for: Comparison of three frailty measures for predicting hospitalization and mortality in the Canadian Longitudinal Study on Aging
Source: Aging Clin Exp Res. 2024 Feb 29;36(1):48. doi: 10.1007/s40520-024-02706-w (PMC10902012; doi:10.1007/s40520-024-02706-w)

**SUPPLEMENTARY MATERIAL**

**Supplementary table 1** Variables used to measure frailty: the grip strength approach.

| **Grip strength** | **Variable used** | **Variable(s) description** | **Grip strength cut-off point** |
| --- | --- | --- | --- |
| **Weakness (average)** | GS_EXAM_AVG_COM | Average grip strength for all trials | **Frail**: Lowest quintile stratified by sex and BMI class |
| **Weakness (max)** | GS_EXAM_MAX_COM | Max grip strength attained on all trials | **Frail**: Lowest quintile stratified by sex and BMI class |

**Supplementary table 2** Variables used to measure frailty in the frailty phenotype approach.

| **Phenotype component** | **Variable(s) used** | **Variable(s) description** | **Phenotype cut-off point** |
| --- | --- | --- | --- |
| **Weight loss** | NUR_weightloss.pheno (constructed based on HWT_DISW_COM, NUR_GLSWT_MCQ, and NUR_WTGL_MCQ) | SCREEN II-AB: How much weight gained or lost | Component present: 6 lbs or more lost in last 6 months for those in BMI class 1 or 2 (under or normal weight) |
| **Weakness (average)** | GS_EXAM_AVG_COM | Average grip strength for all trials | Component present: Lowest quintile stratified by sex and BMI class |
| **Exhaustion** | Exhaustion (constructed based on DEP_GTGO_COM and DEP_FFRT_COM) | Frequency of :1) How often did you feel that you could not "get going"? ; 2) How often did you feel that everything you did was an effort? | Component present: "all the time" or "occasionally" to at least one of the two questions |
| **Slowness** | WLK_TIME_COM | Total time required to complete 4m walk (in seconds) | Component present: Lowest quintile stratified by sex and median height |
| **Low physical activity** | low.PA | Based on the Physical Activity Scale for the Elderly (PASE) questionnaire, total score | Component present: Lowest quintile stratified by sex |
| **Overall Frailty**  **(phenotype approach)** | **NUR_weightloss.pheno, GS_EXAM_AVG_COM, Exhaustion, WLK_TIME_COM, low.PA** | **Number of components present** | **Frail: 3 or more of the 5 components present** |

**Supplementary table 3** Variables used to measure frailty: the Frailty Index (FI) approach.

| **FI Deficit and specific variable used** | | **Variable description** | **Deficit criteria (1: deficit, 0=no deficit)** |
| --- | --- | --- | --- |
| **Chronic conditions** | | | |
|  | CCC_ALLRG_COM | Allergies | 1: have condition, 0: don't have condition |
|  | CCC_ALZH_COM | Dementia or Alzheimer’s disease | 1: have condition, 0: don't have condition |
|  | CCC_AMI_COM | Heart attack or myocardial infarction | 1: have condition, 0: don't have condition |
|  | CCC_ANGI_COM | angina | 1: have condition, 0: don't have condition |
|  | CCC_ANXI_COM | Anxiety disorder | 1: have condition, 0: don't have condition |
|  | CCC_ASTHM_COM | Asthma | 1: have condition, 0: don't have condition |
|  | CCC_BCKP_COM | Back problems excluding fibromyalgia and arthritis | 1: have condition, 0: don't have condition |
|  | CCC_BOWINC_COM | Bowel incontinence | 1: have condition, 0: don't have condition |
|  | CCC_CANC_COM | Cancer | 1: have condition, 0: don't have condition |
|  | CCC_COPD_COM | Emphysema, chronic bronchitis, COPD, or chronic changes in lungs due to smoking | 1: have condition, 0: don't have condition |
|  | CCC_CVA_COM | Stroke or CVA | 1: have condition, 0: don't have condition |
|  | CCC_EPIL_COM | Epilepsy | 1: have condition, 0: don't have condition |
|  | CCC_HBP_COM | High blood pressure or hypertension | 1: have condition, 0: don't have condition |
|  | CCC_HEART_COM | Heart disease (including congestive heart failure, or CHF) | 1: have condition, 0: don't have condition |
|  | CCC_IBDIBS_COM | Bowel disorder | 1: have condition, 0: don't have condition |
|  | CCC_KIDN_COM | Kidney disease or kidney failure | 1: have condition, 0: don't have condition |
|  | CCC_MACDEG_COM | Macular degeneration | 1: have condition, 0: don't have condition |
|  | CCC_MEMPB_COM | Memory problem | 1: have condition, 0: don't have condition |
|  | CCC_MGRN_COM | Migraine headaches | 1: have condition, 0: don't have condition |
|  | CCC_MOOD_COM | Mood disorder | 1: have condition, 0: don't have condition |
|  | CCC_MS_COM | Multiple sclerosis | 1: have condition, 0: don't have condition |
|  | CCC_OAHAND_COM | Osteoarthritis in one or both hands | 1: have condition, 0: don't have condition |
|  | CCC_OAHIP_COM | Osteoarthritis in the hip | 1: have condition, 0: don't have condition |
|  | CCC_OAKNEE_COM | Osteoarthritis in the knee | 1: have condition, 0: don't have condition |
|  | CCC_OSTPO_COM | Osteoporosis | 1: have condition, 0: don't have condition |
|  | CCC_OTHYR_COM | OVER-active thyroid gland (hyperthyroidism) | 1: have condition, 0: don't have condition |
|  | CCC_PARK_COM | Parkinsonism or Parkinson’s Disease | 1: have condition, 0: don't have condition |
|  | CCC_PVD_COM | Peripheral vascular disease or poor circulation in limbs | 1: have condition, 0: don't have condition |
|  | CCC_RA_COM | Rheumatoid arthritis | 1: have condition, 0: don't have condition |
|  | CCC_TIA_COM | Experienced a ministroke or TIA | 1: have condition, 0: don't have condition |
|  | CCC_ULCR_COM | Intestinal or stomach ulcers | 1: have condition, 0: don't have condition |
|  | CCC_URIINC_COM | Urinary incontinence | 1: have condition, 0: don't have condition |
|  | CCC_UTHYR_COM | UNDER-active thyroid gland | 1: have condition, 0: don't have condition |
|  | DIA_DIAB_COM | Diabetes, borderline diabetes or blood sugar is high | 1: have condition, 0: don't have condition |
|  | | | |
| **Activities of daily living and instrumental activities of daily living** | | | |
|  | ADL_ABLAP_COM | OARS scale: Able to take care of appearance | 1: no, 0: yes |
|  | ADL_ABLBD_COM | OARS scale: Able to get out of bed | 1: no, 0: yes |
|  | ADL_ABLBT_COM | OARS scale: Able to take bath | 1: no, 0: yes |
|  | ADL_ABLDR_COM | OARS scale: Able to dress | 1: no, 0: yes |
|  | ADL_ABLFD_COM | OARS scale: Able to feed | 1: no, 0: yes |
|  | ADL_ABLWK_COM | OARS scale: Able to walk | 1: no, 0: yes |
|  | ADL_BATH_COM | OARS scale: Trouble to get in time to bathroom | 1: yes, 0: no |
|  | IAL_ABLGRO_COM | OARS scale: Able to go shopping | 1: no, 0: yes |
|  | IAL_ABLMED_COM | OARS scale: Able to take medicine | 1: no, 0: yes |
|  | IAL_ABLML_COM | OARS scale: Able to prepare meals | 1: no, 0: yes |
|  | IAL_ABLMO_COM | OARS scale: Able to handle money | 1: no, 0: yes |
|  | IAL_ABLTEL_COM | OARS scale: Able to use telephone | 1: no, 0: yes |
|  | IAL_ABLTRV_COM | OARS scale: Able to travel | 1: no, 0: yes |
|  | IAL_ABLWRK_COM | OARS scale: Able to do housework | 1: no, 0: yes |
|  | |  |  |
| **Depression scale** | |  |  |
|  | DEP_BOTR_COM | CES-D 10 scale: Frequency easily bothered | 1: answer "all the time" or "occasionally", |
|  |  |  | 0: answer "sometimes" or "rarely/never" |
|  | DEP_FFRT_COM | CES-D 10 scale: Frequency feel everything is an effort | 1: answer "all the time" or "occasionally", |
|  |  |  | 0: answer "sometimes" or "rarely/never" |
|  | DEP_FLDP_COM | CES-D 10 scale: Frequency feel depressed | 1: answer "all the time" or "occasionally", |
|  |  |  | 0: answer "sometimes" or "rarely/never" |
|  | DEP_FRFL_COM | CES-D 10 scale: Frequency feel fearful or tearful | 1: answer "all the time" or "occasionally", |
|  |  |  | 0: answer "sometimes" or "rarely/never" |
|  | DEP_GTGO_COM | CES-D 10 scale: Frequency feel could not 'get going' | 1: answer "all the time" or "occasionally", |
|  |  |  | 0: answer "sometimes" or "rarely/never" |
|  | DEP_HAPP_COM | CES-D 10 scale: Frequency feel happy | 1: answer "sometimes" or "rarely/never", 0: answer "all the time" or "occasionally" |
|  | DEP_HPFL_COM | CES-D 10 scale: Frequency feel hopeful about the future | 1: answer "sometimes" or "rarely/never", 0: answer "all the time" or "occasionally" |
|  | DEP_LONLY_COM | CES-D 10 scale: Frequency feel lonely | 1: answer "all the time" or "occasionally", |
|  |  |  | 0: answer "sometimes" or "rarely/never" |
|  | DEP_MIND_COM | CES-D 10 scale: Frequency trouble concentrating | 1: answer "all the time" or "occasionally", |
|  |  |  | 0: answer "sometimes" or "rarely/never" |
|  | DEP_RSTLS_COM | CES-D 10 scale: Frequency sleep is restless | 1: answer "all the time" or "occasionally", |
|  |  |  | 0: answer "sometimes" or "rarely/never" |
|  | |  |  |
| **Satisfaction with life** | |  |  |
|  | SLS_DCOND_COM | SWLS: The conditions of my life are excellent - Grouped | 1: answer "Strongly disagree" or "disagree", |
|  |  |  | 0: answer "slightly disagree", "neither agree or disagree" or any agreement |
|  | SLS_DIMP_COM | SWLS: I have gotten the important things I want in life - Grouped | 1: answer "Strongly disagree" or "disagree", |
|  |  |  | 0: answer "slightly disagree", "neither agree or disagree" or any agreement |
|  | SLS_DLIFE_COM | SWLS: Life is close to my ideal - Grouped | 1: answer "Strongly disagree" or "disagree", |
|  |  |  | 0: answer "slightly disagree", "neither agree or disagree" or any agreement |
|  | SLS_DOVER_COM | SWLS: If I could live my life over, I would change almost nothing - Grouped | 1: answer "Strongly disagree" or "disagree", |
|  |  |  | 0: answer "slightly disagree", "neither agree or disagree" or any agreement |
|  | SLS_DSATS_COM | SWLS: I am satisfied with my life - Grouped | 1: answer "Strongly disagree" or "disagree", |
|  |  |  | 0: answer "slightly disagree", "neither agree or disagree" or any agreement |
|  | |  |  |
| **Self-rated health** | |  |  |
|  | GEN_HLTH_COM | Self-rated general health | 1: answer "fair" or "poor”, 0: answer "excellent", "very good" or "good" |
|  | GEN_MNTL_COM | Self-rated mental health | 1: answer "fair" or "poor”, 0: answer "excellent", "very good" or "good" |
|  | GEN_OWNAG_COM | Self-rated healthy aging | 1: answer "fair" or "poor", 0: answer "excellent", "very good" or "good" |
|  | |  |  |
| **Physical activity** | |  |  |
|  | PA2_housework | PASE scale: engaged in home repairs, heavy housework or chores, or light housework over the past 7 days | 1: if answered "no" to all activities in this group, |
|  |  |  | 0: if answered "yes" to any of the activities in this group |
|  | PA2_sport | PASE scale: Participated in outdoor activities or sports (light sports, moderate sports, strenuous sports,lawn work or yard care, outdoor gardening, sweeping the balcony or the stairs, or exercise to increase muscle strength and endurance) over the past 7 days | 1: if answered "no" to all activities in this group, |
|  |  |  | 0: if answered "yes" to any of the activities in this group |
|  | PA2_WALK_MCQ | PASE scale: Frequency of taking a walk outside - past 7 days | 1: answer "never", |
|  |  |  | 0: answer "seldom", "sometimes" or "often" |
|  | |  |  |
| **Nutritional risk** | |  |  |
|  | NUR_APPTT_MCQ | SCREEN II-AB: Describe appetite | 1: answer "poor", |
|  |  |  | 0: answer "very good", "good" or "fair" |
|  | NUR_DRKFLD_MCQ | SCREEN II-AB: Cups of fluid per day | 1: 2 cups or less, |
|  |  |  | 0: more than 2 cups |
|  | NUR_FRTVEG_MCQ | SCREEN II-AB: Servings fruits and vegetables per day | 1: less than two, |
|  |  |  | 0: two or above |
|  | NUR_MLPREP_MCQ | SCREEN II-AB: Experience of meal preparation by oneself | 1: answer "I usually find cooking a chore", |
|  |  |  | 0: answer "I enjoy cooking most of my meals", or "I sometimes find cooking a chore" |
|  | NUR_MLSMN_MCQ | SCREEN II-AB: Meals with someone at least once a day | 1: answer "rarely", or "never", |
|  |  |  | 0: answer "sometimes", "often" or "almost every day" |
|  | NUR_SKPMLS_MCQ | SCREEN II-AB: Skipped meals | 1: answer "almost every day" or "often", 0: answer "sometimes", "rarely", or "never" |
|  | NUR_SWLLFD_MCQ | SCREEN II-AB: Cough, choke pain when swallowing food | 1: answer "almost every day" or "often", 0: answer "sometimes", "rarely", or "never" |
|  | NUR_lost.6lb.ormore | SCREEN II-AB: How much weight gained or lost | 1: 6 or more lbs lost in past 6 months, 0: less than 6 lbs lost |
|  | | | |
| **Physical/performance measures** | | | |
|  | HWT_DBMI_COM | Body Mass Index | 1: BMI<18.5 or >30, |
|  |  |  | 0: BMI between and including 18.5 and 30 |
|  | BP_SYSTOLIC_AVG_COM | Average systolic blood pressure (excluding 1st reading) | 1: systolic BP <=90 or >=140, |
|  |  |  | 0: between 90 and 140 |
|  | SPR_FEV1_TXXX_COM | Forced expiratory volume after 1 second - Median of all trials | 1: lowest quintile stratified by sex and median height, |
|  |  |  | 0: above lowest quintile |
|  | CR_AVG_TIME_COM | Average time for 1 chair rise (in seconds) | 1: highest quintile stratified by sex and BMI class, |
|  |  |  | 0: below highest quintile |
|  | GS_EXAM_AVG_COM | Average grip strength for all trials (in kg) | 1: lowest quintile stratified by sex and BMI class, |
|  |  |  | 0: above lowest quintile |
|  | TUG_TIME_COM | Total time required to complete Timed Get Up and Go (in seconds) | 1: highest quintile stratified by sex and BMI class, |
|  |  |  | 0: below highest quintile |
|  | WLK_TIME_COM | Total time required to complete 4mWalk (in seconds) | 1: highest quintile stratified by sex and median height (greater than, or below), 0: below highest quintile |
|  | BAL_BEST_COM | Best attained time - Standing Balance | 1: lowest quintile stratified by sex and BMI class, |
|  |  |  | 0: above lowest quintile |
|  | | | |
| **Cognitive measures** | | | |
|  | COG_AFT_SCORE_2_COM | AFT - Score 2 - Number of different animals recited in 60 seconds | 1: lowest quintile stratified by sex and education (below or above post-secondary education), 0: above lowest quintile |
|  | COG_MAT_SCORE_COM | MAT - Number of correct consecutive numeric and alphabetical alternations in 30 seconds | 1: lowest quintile stratified by sex and education (below or above post-secondary education), 0: above lowest quintile |
|  | COG_REYI_SCORE_COM | REYI - Number of words (or variants) correctly recalled in 90 seconds - Immediate Recall | 1: lowest quintile stratified by sex and education (below or above post-secondary education), 0: above lowest quintile |
|  | COG_REYII_SCORE_COM | REYII - Number of words (or variants) correctly recalled in 90 seconds - Delayed Recall | 1: lowest quintile stratified by sex and education (below or above post-secondary education), 0: above lowest quintile |
|  | TMT.combined.score | Combined score for time based prospective memory test, including intention to perform, accuracy of response and need of reminders | 1: combined score of three components is below 7, |
|  |  |  | 0: score 7 or above |
|  | PMT.combined.score | Combined score for event based prospective memory test, including intention to perform, accuracy of response and need of reminders | 1: combined score of three components is below 7, |
|  |  |  | 0: score 7 or above |
|  | CRT_MRTWOUT_CORRANS_COM | Mean Reaction Time of correct answers without outliers (ms) | 1: highest quintile stratified by sex and education (below or above post-secondary education), 0: below highest quintile |
|  | FAS_S_SCORE_COM | FAS score - Number of S words recited in 60 seconds | 1: lowest quintile stratified by sex and education (below or above post-secondary education), 0: above lowest quintile |
| **Overall Frailty (Frailty index approach)** | | | |
|  | **All 93 variables presented above** | **FI = Number of deficits present/ Total number of variables** | **Frail: FI > 0.2**  **Not frail: FI ≤ 0.2** |

**Supplementary table 4** List of 35 variables on lifetime history of chronic conditions used to construct the multimorbidity index. The final index was calculated as the sum of the present conditions.

| **Variables used to construct the multimorbidity index** | **Presence** | **Absence** |
| --- | --- | --- |
| Mood disorder | 1 | 0 |
| Anxiety | 1 | 0 |
| Osteoarthritis | 1 | 0 |
| Rheumatoid arthritis | 1 | 0 |
| Other type of arthritis | 1 | 0 |
| Asthma | 1 | 0 |
| COPD | 1 | 0 |
| Hypertension | 1 | 0 |
| Heart disease | 1 | 0 |
| Angina | 1 | 0 |
| Myocardial infarction | 1 | 0 |
| Peripheral vascular disease | 1 | 0 |
| Stroke or TIA | 1 | 0 |
| Gastrointestinal ulcer | 1 | 0 |
| Bowel disorder | 1 | 0 |
| Kidney disease or failure | 1 | 0 |
| Hypothyroidism | 1 | 0 |
| Hyperthyroidism | 1 | 0 |
| Diabetes | 1 | 0 |
| Osteoporosis | 1 | 0 |
| Cataracts | 1 | 0 |
| Glaucoma | 1 | 0 |
| Macular degeneration | 1 | 0 |
| Epilepsy | 1 | 0 |
| Parkinson’s disease | 1 | 0 |
| Multiple sclerosis | 1 | 0 |
| Migraine | 1 | 0 |
| Allergies | 1 | 0 |
| Skin cancer: melanoma | 1 | 0 |
| Skin cancer: non-melanoma | 1 | 0 |
| Solid cancer | 1 | 0 |
| Haematological and soft cancer | 1 | 0 |
| Ill-defined cancer | 1 | 0 |
| Current smoker | 1 | 0 |
| Regular drinker | 1 | 0 |

**Supplementary table 5** Selected characteristics of study participants in the unimputed and pooled imputed dataset.

| **Selected characteristics** | **Unimputed dataset** | **Imputed dataset^1^** |
| --- | --- | --- |
|  | **N = 28,527** | **N = 28,527** |
|  | **n (%)** | **n (%)** |
| **Average grip strength** |  |  |
| Frail | 5,527 (19.4) | 5,697 (20.0) |
| Non-frail | 22,143 (77.6) | 22,830 (80.0) |
| Missing | 857 (3.0) | 0 (0.0) |
| **Max grip strength** |  |  |
| Frail | 5,526 (19.4) | 5,697 (20.0) |
| Non-frail | 22,144 (77.6) | 22,830 (80.0) |
| Missing | 857 (3.0) | 0 (0.0) |
| **Frailty index^2^** |  |  |
| Frail | 2,250 (7.9) | 2,307 (8.1) |
| Non-frail | 26,277 (92.1) | 26,220 (91.9) |
| Missing | 0 (0.0) | 0 (0.0) |
| **Frailty phenotype** |  |  |
| Frail | 1,551 (5.4) | 1,708 (6.0) |
| Non-frail | 25,613 (89.8) | 26,819 (94.0) |
| Missing | 1,363 (4.8) | 0 (0.0) |
| **Hospitalization** |  |  |
| Hospitalized | 2,320 (8.1) | 2,634 (9.2) |
| Not hospitalized | 24,020 (84.2) | 25,893 (90.8) |
| Missing | 2,187 (7.7) | 0 (0.0) |
| **Mortality** |  |  |
| Dead | 505 (1.8) | 505 (1.8) |
| Alive | 28,022 (98.2) | 28,022 (98.2) |
| Missing | 0 (0.0) | 0 (0.0) |
| **Multimorbidity index** |  |  |
| Mean (SD) | 3.9 (2.3) | 4.0 (2.4) |
| Missing | 3,274 (11.5) | 0 (0.0) |
| **Age (year)** |  |  |
| Mean (SD) | 62.8 (10.2) | 62.8 (10.2) |
| Missing | 0 (0.0) | 0 (0.0) |
| **Sex** |  |  |
| Male | 14,269 (50.0) | 14,269 (50.0) |
| Female | 14,258 (50.0) | 14,258 (50.0) |
| Missing | 0 (0.0) | 1. (0.0) |

SD: standard deviation

1. Average of the 15 imputed datasets.
2. The difference between the unimputed and imputed dataset is due to the addition of previously missing deficit variables in the calculation of the frailty index.

**Supplementary table 6** Adjusted ORs (95% CIs) and c-statistics (95% CIs) for the association between different indicators of frailty in older adults and hospitalization, stratified by sex.^1^

| **Frailty indicators** | **Male sex** | | **Female sex** | |
| --- | --- | --- | --- | --- |
|  | **OR**  **(95% CI)** | **C-statistic**  **(95% CI)** | **OR**  **(95% CI)** | **C-statistic**  **(95% CI)** |
| **Grip strength** |  |  |  |  |
| Average |  |  |  |  |
| Continuous^2,3^ | 0.97 (0.90-1.05) | 0.68 (0.66-0.69) | 0.95 (0.88-1.03) | 0.66 (0.64-0.68) |
| frail vs. non-frail^5^ | 1.08 (0.91-1.27) | 0.68 (0.66-0.69) | 1.00 (0.83-1.21) | 0.66 (0.64-0.68) |
| Max |  |  |  |  |
| Continuous^2,3^ | 0.97 (0.90-1.04) | 0.68 (0.66-0.69) | 0.94 (0.86-1.02) | 0.66 (0.64-0.68) |
| frail vs. non-frail^5^ | 1.11 (0.94-1.32) | 0.68 (0.66-0.69) | 1.01 (0.84-1.22) | 0.66 (0.64-0.68) |
| **Frailty phenotype** |  |  |  |  |
| Continuous^2,4^ | 1.23 (1.16-1.31) | 0.69 (0.67-0.70) | 1.22 (1.14-1.31) | 0.67 (0.65-0.68) |
| frail vs. non-frail^6^ | 1.72 (1.36-2.20) | 0.68 (0.67-0.70) | 1.60 (1.26-2.04) | 0.66 (0.65-0.68) |
| **Frailty index** |  |  |  |  |
| Continuous^2,4^ | 1.43 (1.33-1.55) | 0.70 (0.68-0.71) | 1.48 (1.37-1.61) | 0.68 (0.66-0.70) |
| frail vs. non-frail^7^ | 1.87 (1.47-2.39) | 0.68 (0.67-0.70) | 1.86 (1.51-2.29) | 0.67 (0.65-0.68) |

OR: odds ratio; CI: confidence interval.

1. Models were adjusted for the multimorbidity index, age, and sex.

2. Standardized using the z-score method.

3. Lower values for grip strength indicates higher degree of frailty.

4. Higher values for the frailty phenotype and index indicate higher degree of frailty.

5. Frail: Lowest quintile stratified by sex and BMI class. Non-frail: All other higher quantiles.

6. Frail: 3 or more of the five phenotype components present. Non-frail: 0 to 2 of the five phenotype components present.

7. Frail: FI > 0.2. Non-frail: FI ≤ 0.2

**Supplementary table 7** Adjusted ORs (95% CIs) and c-statistics (95% CIs) for the association between different indicators of frailty in older adults and mortality, stratified by sex.^1^

| **Frailty indicators** | **Male sex** | | **Female sex** | |
| --- | --- | --- | --- | --- |
|  | **OR**  **(95% CI)** | **C-statistic**  **(95% CI)** | **OR**  **(95% CI)** | **C-statistic**  **(95% CI)** |
| **Grip strength** |  |  |  |  |
| Average |  |  |  |  |
| Continuous^2,3^ | 0.66 (0.56-0.78) | 0.80 (0.78-0.81) | 0.82 (0.67-1.00) | 0.77 (0.75-0.79) |
| frail vs. non-frail^5^ | 1.61 (1.18-2.21) | 0.79 (0.77-0.81) | 1.33 (0.92-1.94) | 0.77 (0.75-0.79) |
| Max |  |  |  |  |
| Continuous^2,3^ | 0.66 (0.56-0.78) | 0.79 (0.78-0.81) | 0.81 (0.66-0.99) | 0.77 (0.75-0.80) |
| frail vs. non-frail^5^ | 1.65 (1.20-2.26) | 0.79 (0.77-0.81) | 1.49 (1.02-2.17) | 0.77 (0.75-0.80) |
| **Frailty phenotype** |  |  |  |  |
| Continuous^2,4^ | 1.72 (1.52-1.95) | 0.80 (0.78-0.82) | 1.82 (1.57-2.11) | 0.78 (0.75-0.80) |
| frail vs. non-frail^6^ | 3.23 (2.19-4.77) | 0.79 (0.78-0.81) | 3.36 (2.20- 5.14) | 0.77 (0.75-0.80) |
| **Frailty index** |  |  |  |  |
| Continuous^2,4^ | 1.77 (1.54-2.04) | 0.81 (0.79-0.83) | 1.96 (1.66-2.32) | 0.79 (0.76-0.81) |
| frail vs. non-frail^7^ | 3.19 (2.10-4.83) | 0.80 (0.78-0.82) | 3.54 (2.32-5.41) | 0.78 (0.75-0.80) |

OR: odds ratio; CI: confidence interval.

1. Models were adjusted for the multimorbidity index, age, and sex.

2. Standardized using the z-score method.

3. Lower values for grip strength indicates higher degree of frailty.

4. Higher values for the frailty phenotype and index indicate higher degree of frailty.

5. Frail: Lowest quintile stratified by sex and BMI class. Non-frail: All other higher quantiles.

6. Frail: 3 or more of the five phenotype components present. Non-frail: 0 to 2 of the five phenotype components present.

7. Frail: FI > 0.2. Non-frail: FI ≤ 0.2

**Supplementary table 8** Adjusted ORs (95% CIs) and c-statistics (95% CIs) for the association between different indicators of frailty in the adults aged 65 years and older and hospitalization or mortality, in both sexes combined.^1^

| **Frailty indicators** | **Hospitalization** | | **Mortality** | |
| --- | --- | --- | --- | --- |
|  | **OR**  **(95% CI)** | **C-statistic**  **(95% CI)** | **OR**  **(95% CI)** | **C-statistic**  **(95% CI)** |
| **Grip strength** |  |  |  |  |
| Average |  |  |  |  |
| Continuous^2,3^ | 0.95 (0.87 - 1.03) | 0.67 (0.65 - 0.68) | 0.70 (0.57 - 0.87) | 0.68 (0.00 - 0.70) |
| frail vs. non-frail^5^ | 1.04 (0.91 - 1.19) | 0.67 (0.65 - 0.68) | 1.39 (1.03 - 1.86) | 0.68 (0.00 - 1.39) |
| Max |  |  |  |  |
| Continuous^2,3^ | 0.93 (0.86 - 1.01) | 0.67 (0.65 - 0.68) | 0.71 (0.57 - 0.88) | 0.68 (0.00 - 0.71) |
| frail vs. non-frail^5^ | 1.06 (0.93 - 1.22) | 0.67 (0.65 - 0.68) | 1.47 (1.09 - 1.96) | 0.68 (0.00 - 1.47) |
| **Frailty phenotype** |  |  |  |  |
| Continuous^2,4^ | 1.20 (1.14 - 1.26) | 0.67 (0.66 - 0.69) | 1.77 (1.59 - 1.98) | 0.69 (0.00 - 1.77) |
| frail vs. non-frail^6^ | 1.77 (1.44 - 2.16) | 0.67 (0.66 - 0.68) | 3.43 (2.43 - 4.77) | 0.68 (0.00 - 3.43) |
| **Frailty index** |  |  |  |  |
| Continuous^2,4^ | 1.46 (1.38 - 1.55) | 0.69 (0.67 - 0.70) | 1.73 (1.52 - 1.98) | 0.70 (0.00 - 1.73) |
| frail vs. non-frail^7^ | 1.85 (1.54 - 2.23) | 0.67 (0.66 - 0.68) | 2.61 (1.78 - 3.78) | 0.68 (0.00 - 2.61) |

OR: odds ratio; CI: confidence interval.

1. Models were adjusted for the multimorbidity index, age, and sex.

2. Standardized using the z-score method.

3. Lower values for grip strength indicates higher degree of frailty.

4. Higher values for the frailty phenotype and index indicate higher degree of frailty.

5. Frail: Lowest quintile stratified by sex and BMI class. Non-frail: All other higher quantiles.

6. Frail: 3 or more of the five phenotype components present. Non-frail: 0 to 2 of the five phenotype components present.

7. Frail: FI > 0.2. Non-frail: FI ≤ 0.2

**Supplementary table 9** Age and sex adjusted ORs (95% CIs) and c-statistics (95% CIs) for the association between different indicators of frailty in older adults and hospitalization or mortality in both sexes combined.^1^

| **Frailty indicators** | **Hospitalization** | | **Mortality** | |
| --- | --- | --- | --- | --- |
|  | **OR**  **(95% CI)** | **C-statistic**  **(95% CI)** | **OR**  **(95% CI)** | **C-statistic**  **(95% CI)** |
| **Grip strength** |  |  |  |  |
| Average |  |  |  |  |
| Continuous^2,3^ | 0.89 (0.83 - 0.96) | 0.64 (0.63 - 0.65) | 0.58 (0.49 - 0.69) | 0.77 (0.75 - 0.79) |
| frail vs. non-frail^5^ | 1.13 (1.00 - 1.27) | 0.64 (0.63 - 0.65) | 1.63 (1.28 - 2.07) | 0.76 (0.74 - 0.78) |
| Max |  |  |  |  |
| Continuous^2,3^ | 0.88 (0.81 - 0.95) | 0.64 (0.63 - 0.65) | 0.57 (0.48 - 0.68) | 0.77 (0.75 - 0.79) |
| frail vs. non-frail^5^ | 1.15 (1.02 - 1.29) | 0.64 (0.63 - 0.65) | 1.73 (1.36 - 2.20) | 0.76 (0.74 - 0.78) |
| **Frailty phenotype** |  |  |  |  |
| Continuous^2,4^ | 1.32 (1.26 - 1.38) | 0.68 (0.67 - 0.69) | 1.89 (1.73 - 2.07) | 0.80 (0.78 - 0.82) |
| frail vs. non-frail^6^ | 2.04 (1.73 - 2.39) | 0.65 (0.64 - 0.66) | 4.06 (3.07 - 5.39) | 0.77 (0.75 - 0.79) |
| **Frailty index** |  |  |  |  |
| Continuous^2,4^ | 1.55 (1.49 - 1.62) | 0.69 (0.68 - 0.70) | 1.87 (1.72 - 2.02) | 0.81 (0.79 - 0.83) |
| frail vs. non-frail^7^ | 2.75 (2.39 - 3.15) | 0.66 (0.65 - 0.67) | 4.59 (3.59 - 5.88) | 0.79 (0.77 - 0.81) |

OR: odds ratio; CI: confidence interval.

1. Models were adjusted for the multimorbidity index, age, and sex.

2. Standardized using the z-score method.

3. Lower values for grip strength indicates higher degree of frailty.

4. Higher values for the frailty phenotype and index indicate higher degree of frailty.

5. Frail: Lowest quintile stratified by sex and BMI class. Non-frail: All other higher quantiles.

6. Frail: 3 or more of the five phenotype components present. Non-frail: 0 to 2 of the five phenotype components present.

7. Frail: FI > 0.2. Non-frail: FI ≤ 0.2

**Supplementary table 10** Complete case analysis: Adjusted ORs (95% CIs) and c-statistics (95% CIs) for the association between different indicators of frailty in older adults and hospitalization or mortality, in both sexes combined.^1^

| **Frailty indicators** | **Hospitalization** | | **Mortality** | |
| --- | --- | --- | --- | --- |
|  | **OR**  **(95% CI)** | **C-statistic**  **(95% CI)** | **OR**  **(95% CI)** | **C-statistic**  **(95% CI)** |
| **Grip strength** |  |  |  |  |
| Average |  |  |  |  |
| Continuous^2,3^ | 0.87 (0.78 - 0.97) | 0.63 (0.61 - 0.64) | 0.71 (0.58 - 0.88) | 0.73 (0.68 - 0.71) |
| frail vs. non-frail^5^ | 1.04 (0.89 - 1.21) | 0.62 (0.61 - 0.64) | 1.38 (1.05 - 1.83) | 0.70 (0.67 - 0.73) |
| Max |  |  |  |  |
| Continuous^2,3^ | 0.85 (0.76 - 0.95) | 0.63 (0.61 - 0.64) | 0.71 (0.57 - 0.88) | 0.70 (0.68 - 0.73) |
| frail vs. non-frail^5^ | 1.10 (0.94 - 1.28) | 0.63 (0.61 - 0.64) | 1.49 (1.12 - 1.98) | 0.70 (0.68 - 0.73) |
| **Frailty phenotype** |  |  |  |  |
| Continuous^2,4^ | 1.30 (1.21 - 1.40) | 0.64 (0.63 - 0.66) | 1.74 (1.53 - 1.98) | 0.72 (0.69 - 0.75) |
| frail vs. non-frail^6^ | 1.72 (1.41 - 2.11) | 0.63 (0.62 - 0.65) | 2.97 (2.17 - 4.06) | 0.71 (0.68 - 0.73) |
| **Frailty index** |  |  |  |  |
| Continuous^2,4^ | 1.55 (1.42 - 1.69) | 0.66 (0.64 - 0.67) | 1.95 (1.69 - 2.25) | 0.74 (0.72 - 0.77) |
| frail vs. non-frail^7^ | 1.89 (1.52 - 2.34) | 0.64 (0.62 - 0.65) | 3.22 (2.26 - 4.57) | 0.72 (0.69 - 0.74) |

OR: odds ratio; CI: confidence interval.

1. Models were adjusted for the multimorbidity index, age, and sex.

2. Standardized using the z-score method.

3. Lower values for grip strength indicates higher degree of frailty.

4. Higher values for the frailty phenotype and index indicate higher degree of frailty.

5. Frail: Lowest quintile stratified by sex and BMI class. Non-frail: All other higher quantiles.

6. Frail: 3 or more of the five phenotype components present. Non-frail: 0 to 2 of the five phenotype components present.

7. Frail: FI > 0.2. Non-frail: FI ≤ 0.2

| **Supplementary table 11** Comparison of participants with and without medical contraindications for the grip strength or the 4-minute walk assessments. | | | | | | | | | |
| --- | --- | --- | --- | --- | --- | --- | --- | --- | --- |
| **Distribution of continuous variables in participants with and without medical contraindications for the grip strength or the 4-minute walk assessments** | | | | | | | | | |
| Continuous variables | min | | Q1 | median | | mean | Q3 | max | |
| Frailty index (in %) |  | |  |  | |  |  |  | |
| No contraindication | 0 | | 4.55 | 7.96 | | 9.63 | 12.5 | 67.86 | |
| with contraindication | 0 | | 9.1 | 13.6 | | 15.3 | 20 | 55 | |
| Age (in years) |  | |  |  | |  |  |  | |
| No contraindication | 45 | | 54 | 62 | | 62.8 | 70 | 86 | |
| with contraindication | 45 | | 59 | 66 | | 66.2 | 74 | 86 | |
| Comorbidity (number of chronic conditions present) |  | |  |  | |  |  |  | |
| No contraindication | 0 | | 2 | 3 | | 3.9 | 5 | 19 | |
| with contraindication | 0 | | 4 | 5 | | 5.5 | 7 | 18 | |
| **Distribution of sex in participants with and without medical contraindications for the grip strength or the 4-minute walk assessments** | | | | | | | | |  |
| Presence of contraindications | | Male n(%) | | | Female n(%) | | | |  |
| No contraindication | | 14,269 (50.0) | | | 14,258 (50.0) | | | |  |
| with contraindication | | 1,061 (67.7) | | | 508 (32.3) | | | |  |
| **Distribution of frailty based on the FI in participants with and without medical contraindications for the grip strength or the 4-minute walk assessments** | | | | | | | | |  |
| Presence of contraindications | | FI: Frail n(%) | | | FI: Not-frail n(%) | | | |  |
| No contraindication | | 22,50 (7.9) | | | 26,277 (92.1) | | | |  |
| with contraindication | | 387 (24.7) | | | 1,182 (75.3) | | | |  |

**Supplementary fig. 1** Flow chart of CLSA participants who met the inclusion/exclusion criteria for the present study.

Included in the analysis

28,527 participants

Full CLSA cohort

(n=51,338 participants)

Comprehensive cohort

(n=30,097 participants)

- Interview
- Physical assessments
- Biosample collections

Excluded

Tracking cohort (n=21,241 participants)

- Interview only

Excluded

Participants (n=1,570) who were

- Unable to complete the grip strength assessment (n=1,456) or the 4-minute walk assessment due to medical contraindications (n=113)
- Pregnant at the time of the baseline interview and thus was unable to be assigned a comparable BMI class (n=1)

**Supplementary fig. 2** Plot of the ORs (95% CIs) for the association between quartile versions of the frailty measurements and hospitalization.


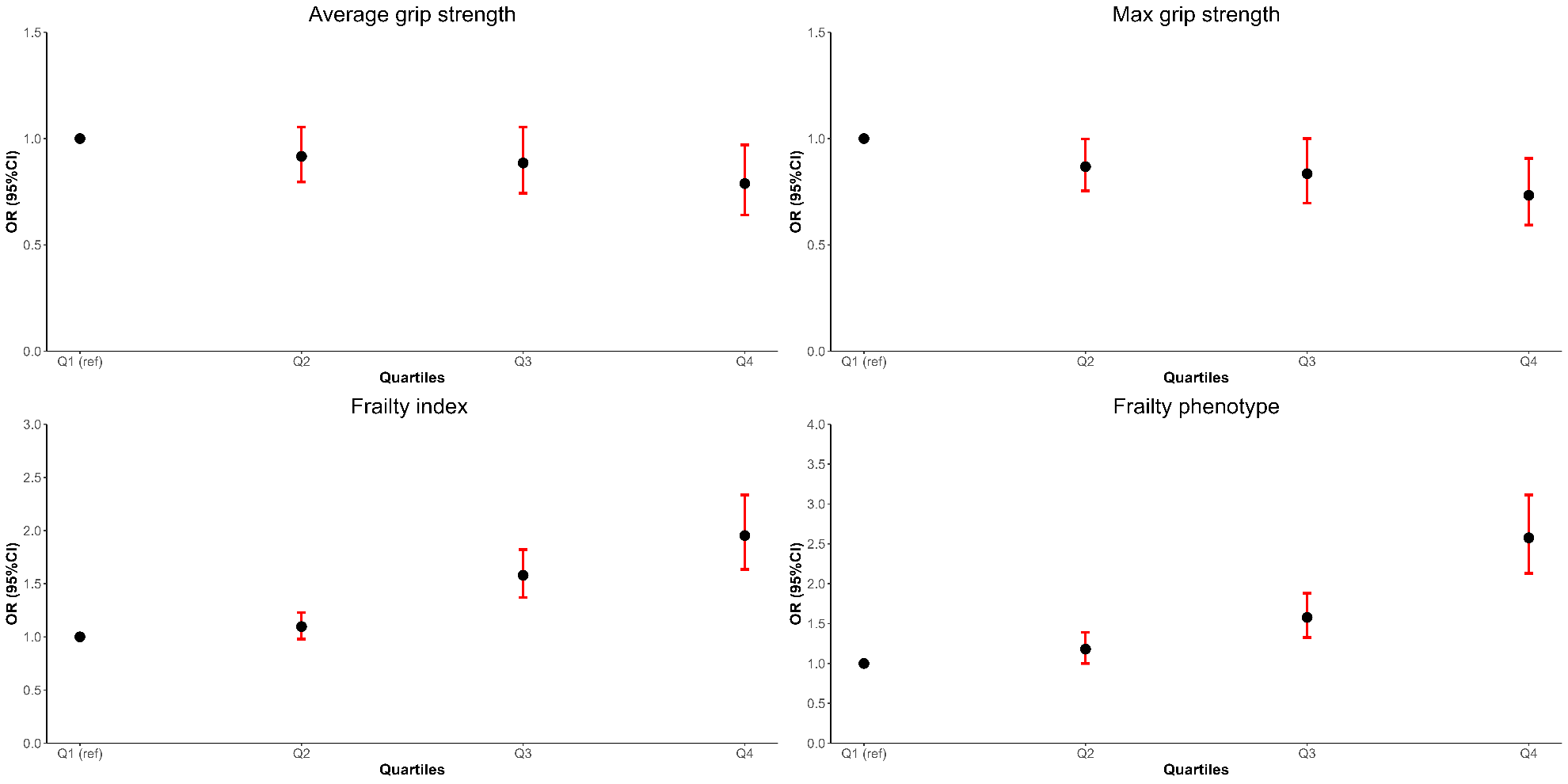


**Supplementary fig. 3** Plot of the ORs (95% CIs) for the association between quartile versions of the frailty measurements and mortality.


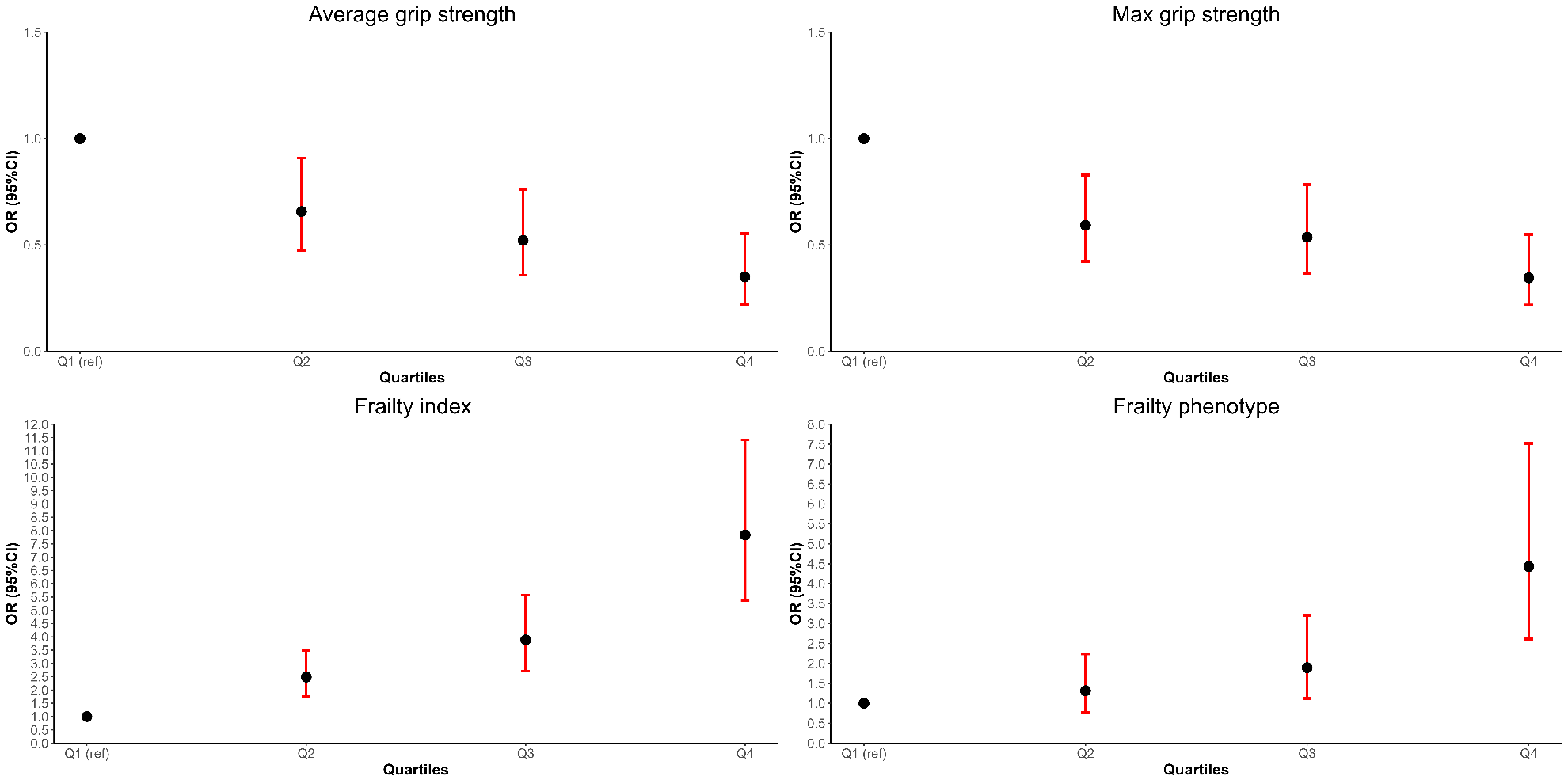


**Supplementary fig. 4** Calibration plots for the continuous hospitalization models in male sex.


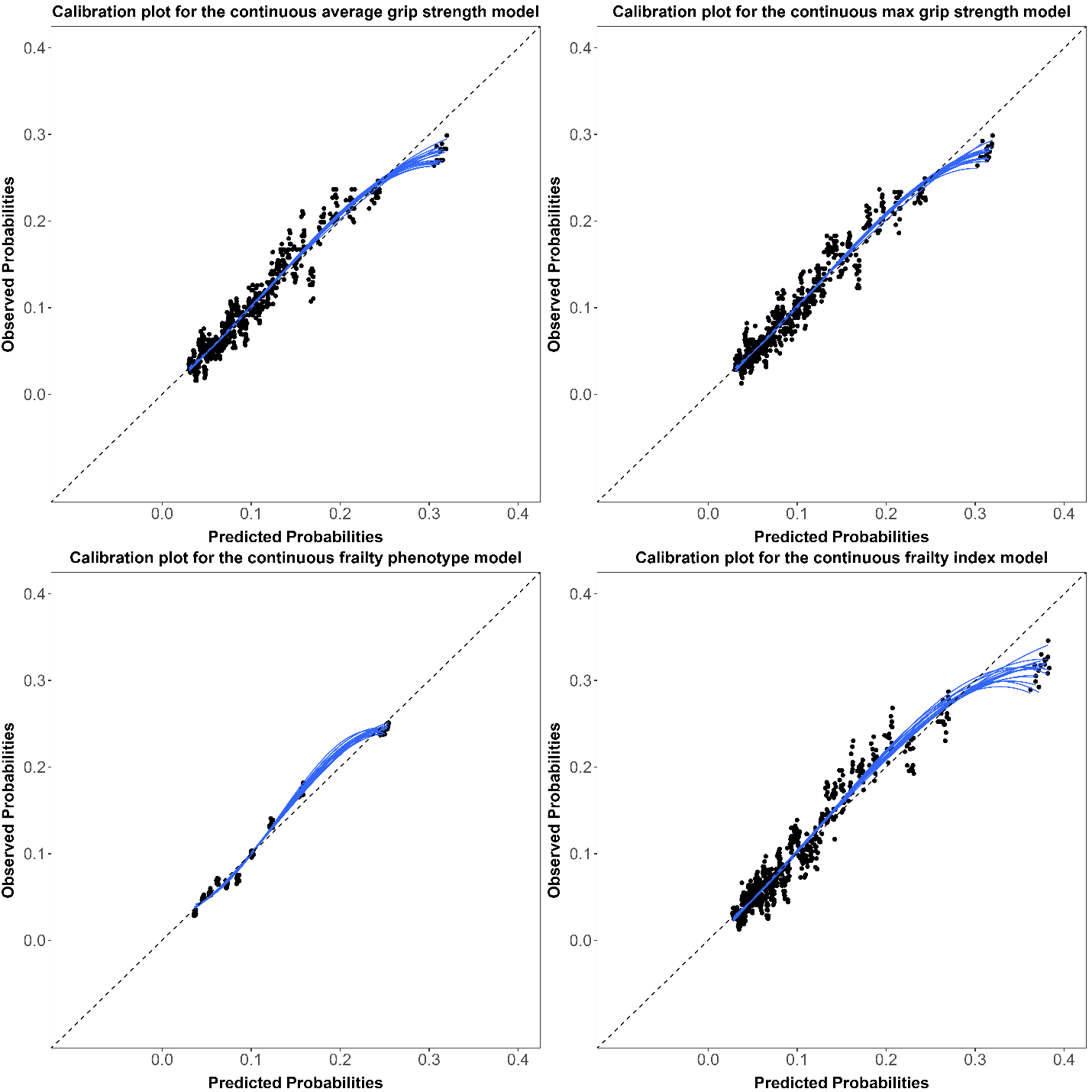


**Supplementary fig. 5** Calibration plots for the continuous hospitalization models in female sex.


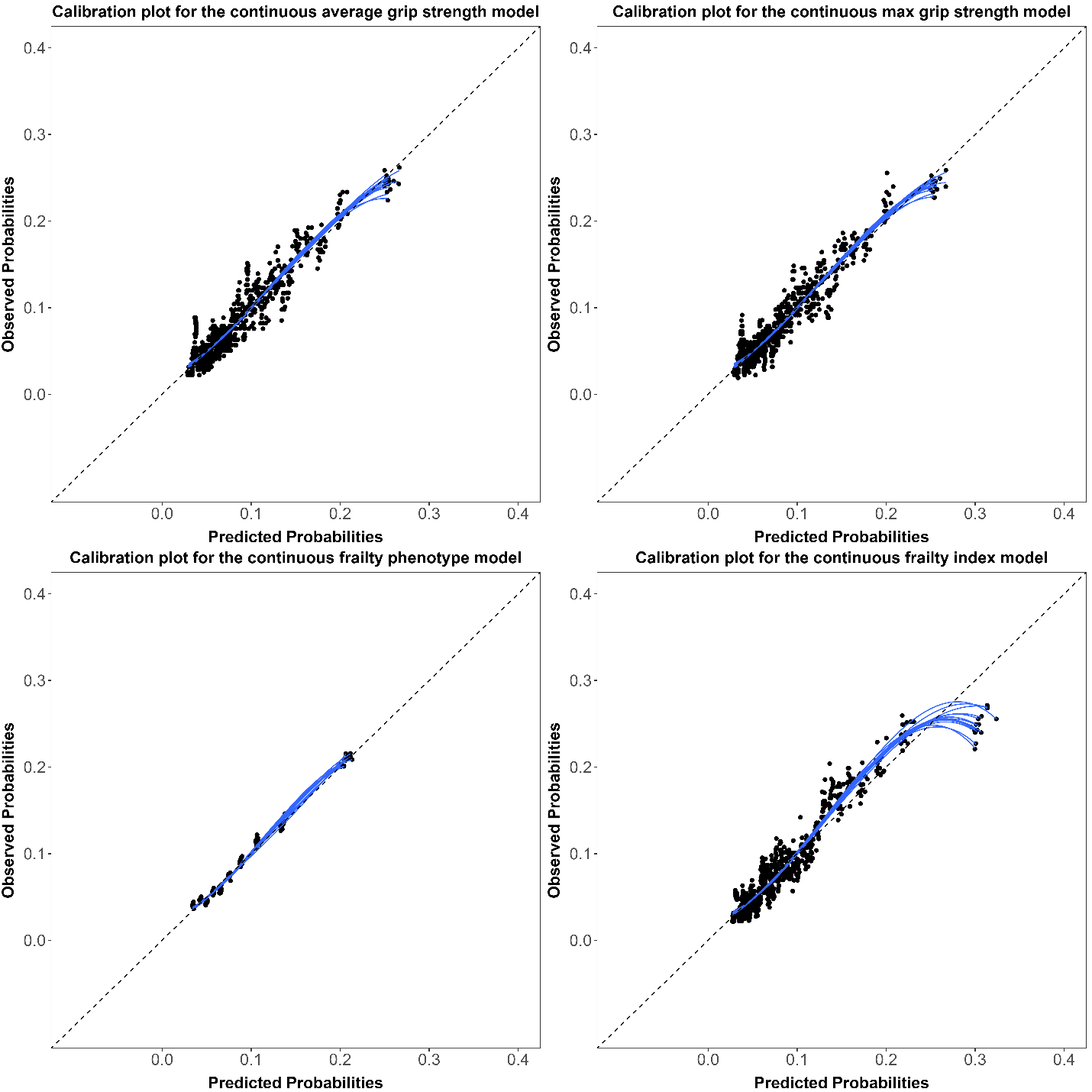


**Supplementary fig. 6** Calibration plots for the binary hospitalization models in male sex.


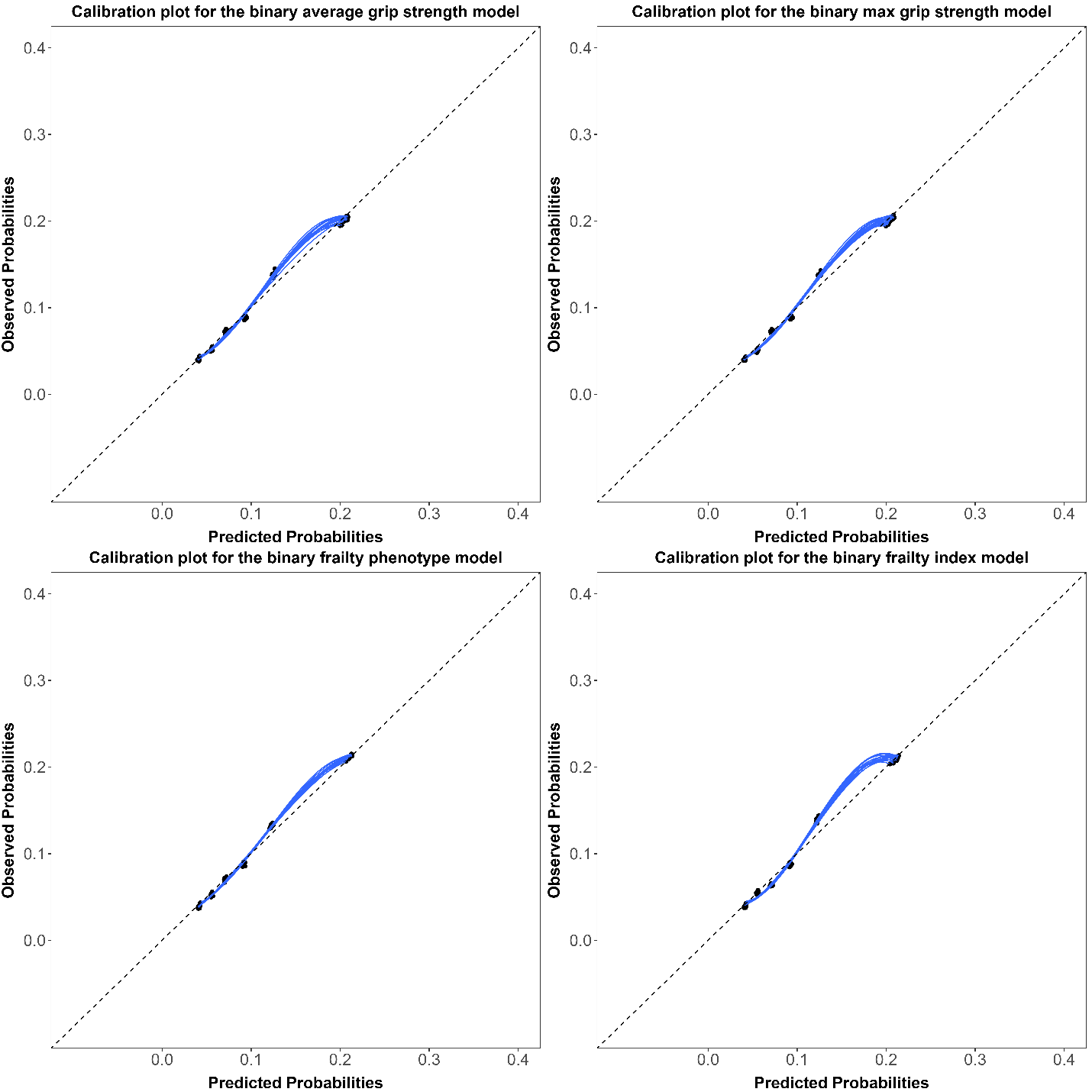


**Supplementary fig. 7** Calibration plots for the binary hospitalization models in female sex.


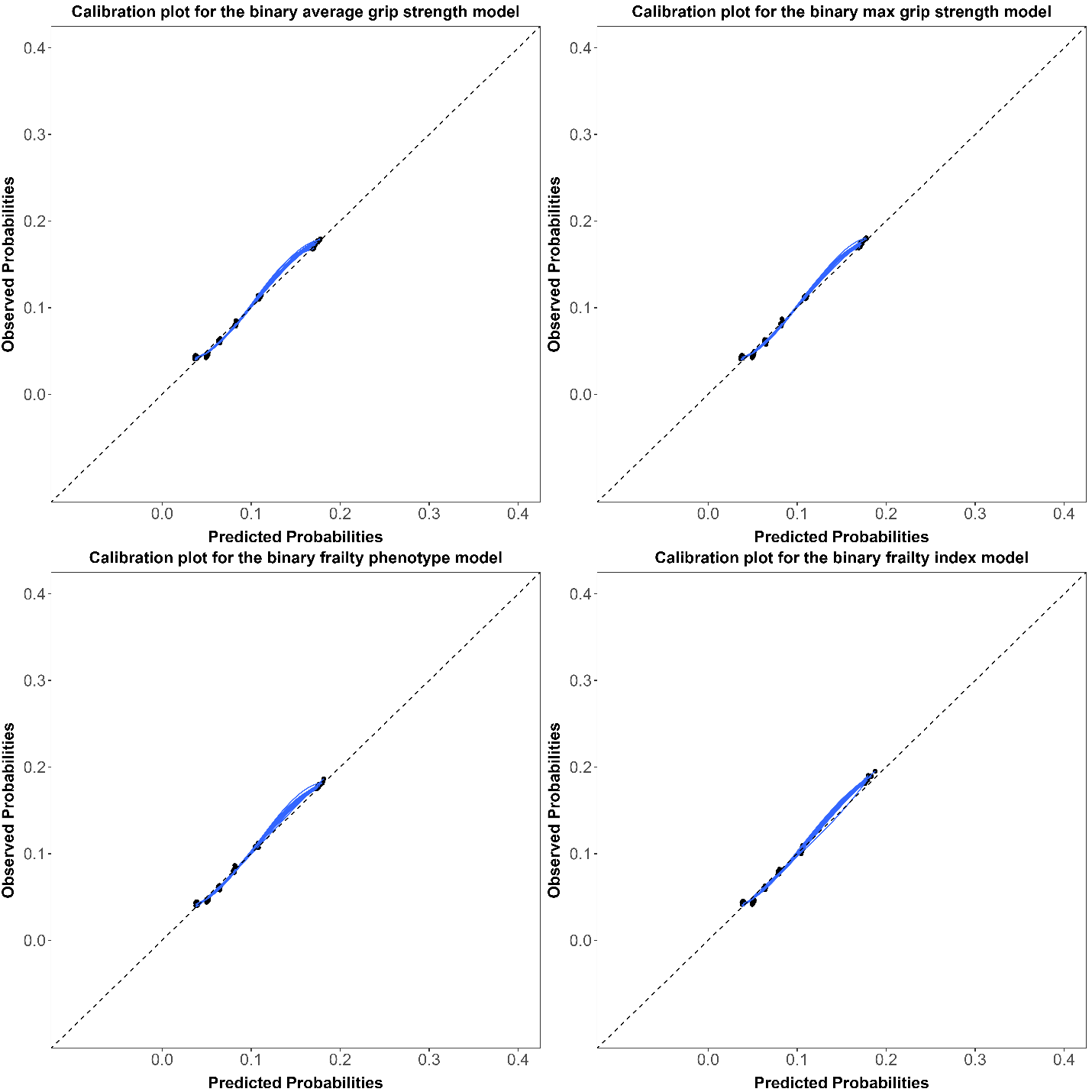


**Supplementary fig. 8** Calibration plots for the continuous mortality models in male sex.


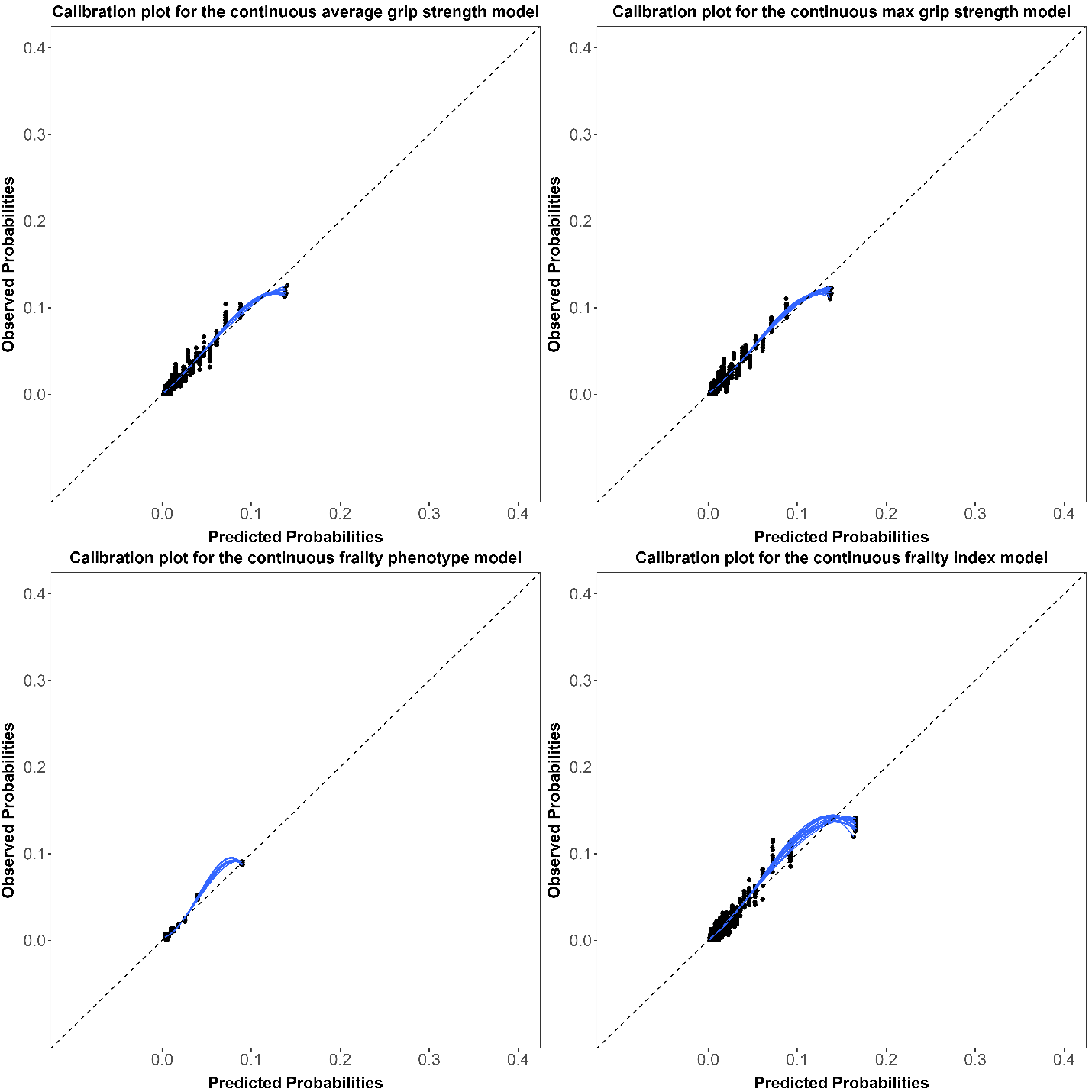


**Supplementary fig. 9** Calibration plots for the continuous mortality models in female sex.


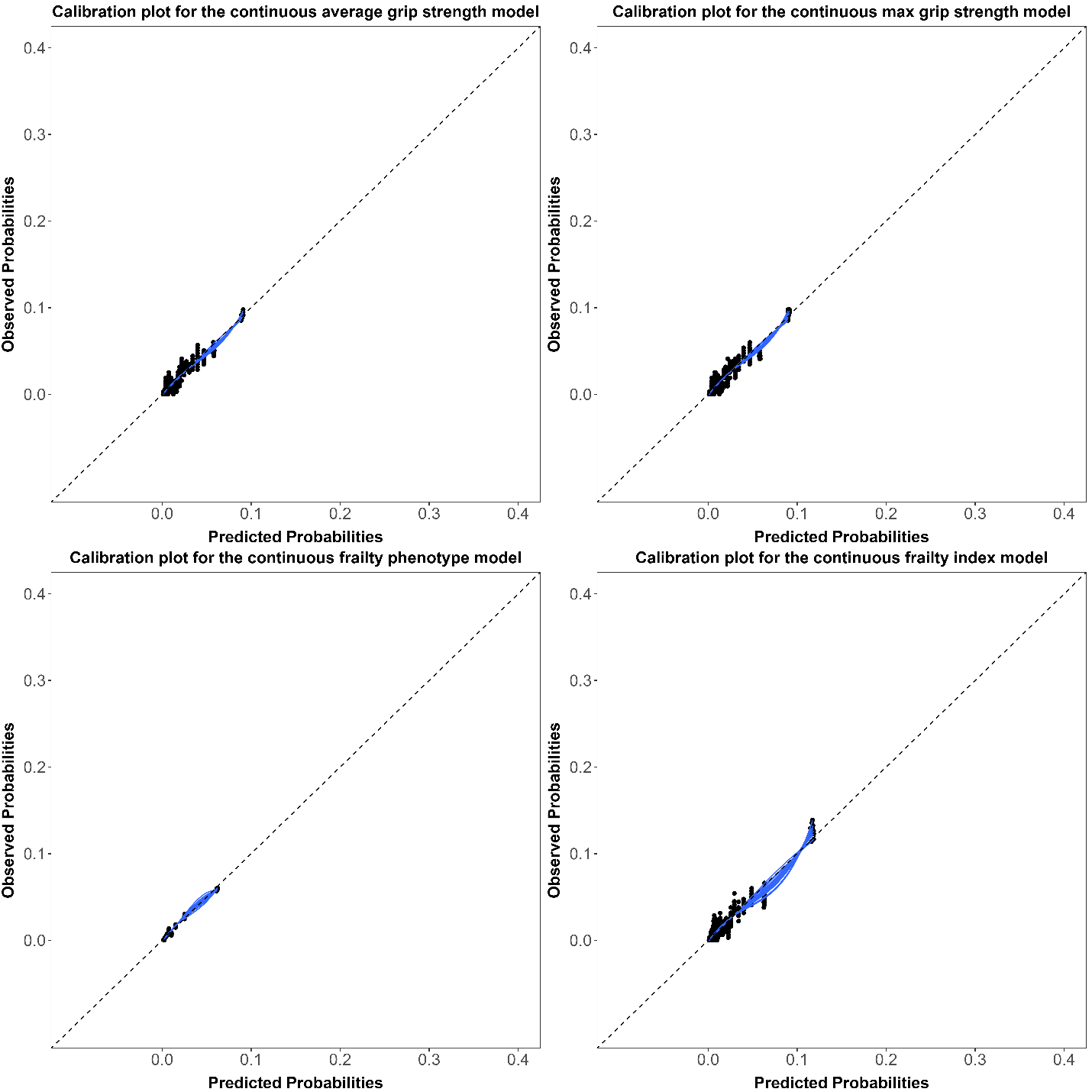


**Supplementary fig. 10** Calibration plots for the binary mortality models in male sex.


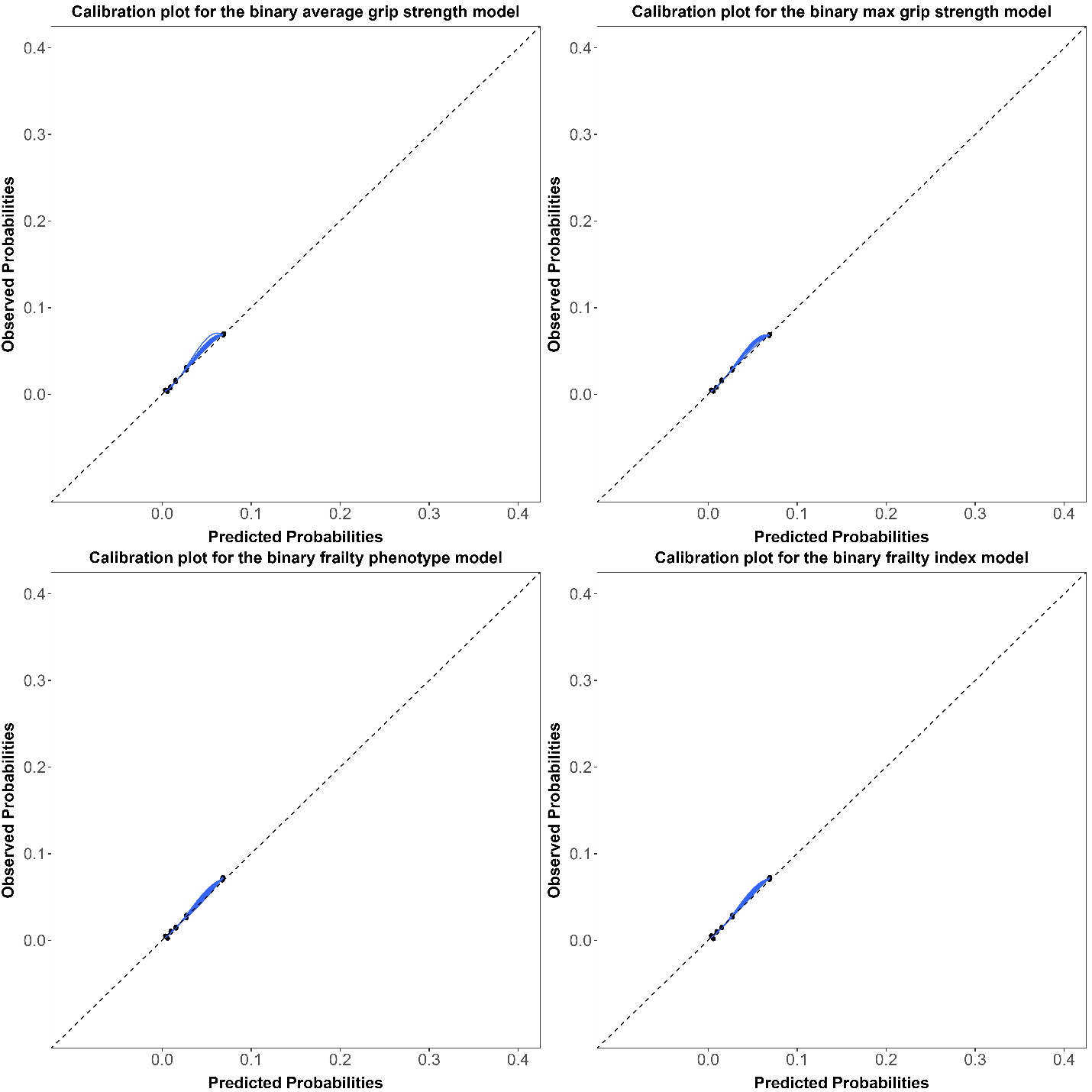


**Supplementary fig. 11** Calibration plots for the binary mortality models in female sex.


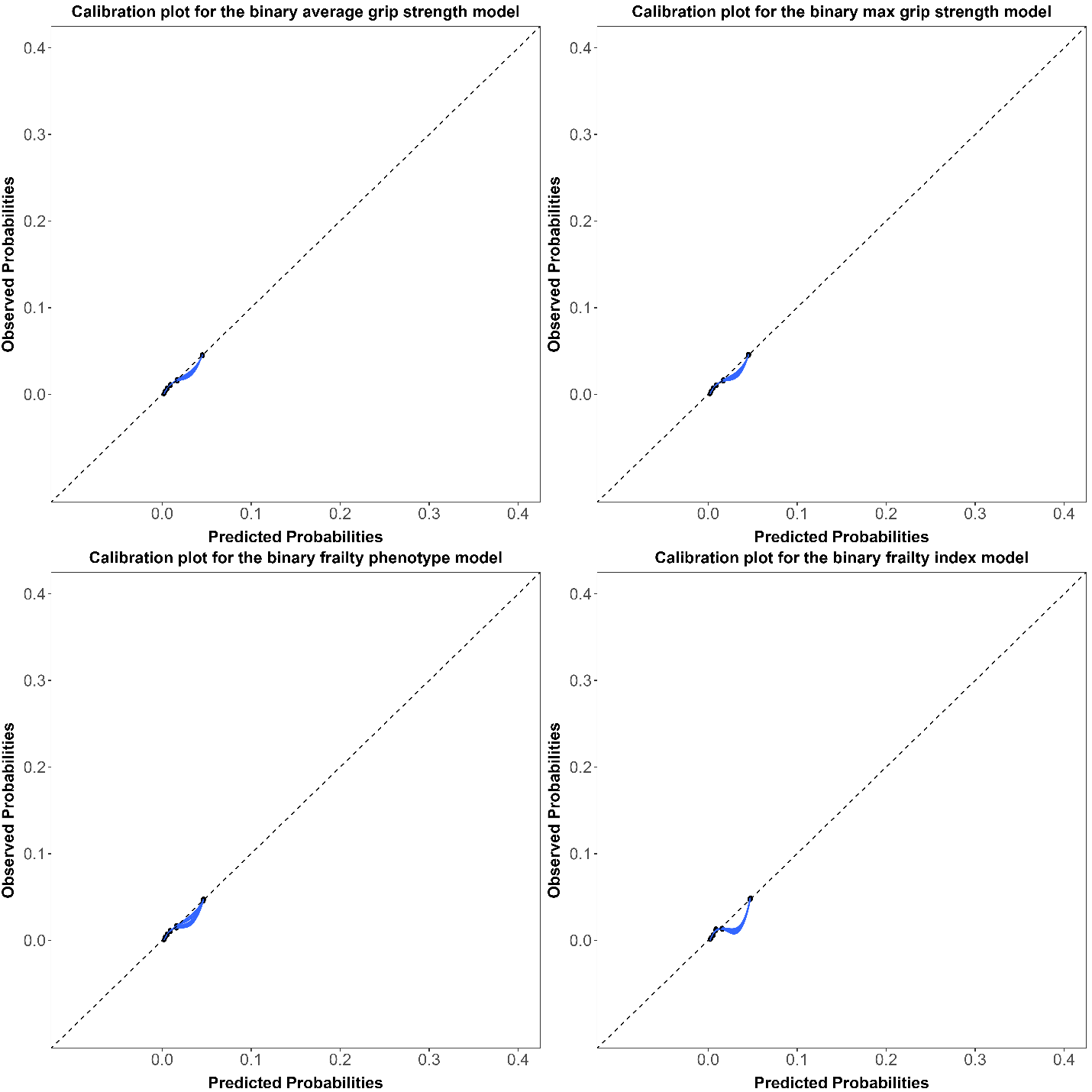

Supplement: Supplementary file 1 — Supplementary file1 (DOCX 885 KB) [file 40520_2024_2706_MOESM1_ESM.docx]
